# METTL3/RBM15 augments the stability of Kdm6b mRNA and promotes STAT1-mediated macrophage activation and atherosclerosis

**DOI:** 10.1038/s12276-025-01594-y

**Published:** 2025-12-22

**Authors:** Ning Huangfu, Fang Li, Chenqiu Wang, Shouyi Jin, Xiaoya Zheng, Yingsong Wang, Tianxiang Fang, Jiaxi Shen, Yanan Yu, Liguo Jian, Datun Qi, Wenting Zhao, Dongdong Jian

**Affiliations:** 1 Department of Cardiology, The First Affiliated Hospital of Ningbo University, Ningbo, China; 2 Department of Cardiology, Key Laboratory of Precision Medicine for Atherosclerotic Diseases of Zhejiang Province, Ningbo, China; 3 Clinical Medicine Research Centre for Cardiovascular Disease of Ningbo, Ningbo, China; 4 Department of Cardiology, The Second Affiliated Hospital of Zhengzhou University, Zhengzhou, China; 5 Tianjian Laboratory of Advanced Biomedical Sciences, Institute of Advanced Biomedical Sciences, Zhengzhou University, Zhengzhou, China; 6 Zhengzhou Key Laboratory of Cardiovascular Aging, Henan Province Key Laboratory for Prevention and Treatment of Coronary Heart Disease, National Health Commission Key Laboratory of Cardiovascular Regenerative Medicine, Central China Fuwai Hospital of Zhengzhou University, Fuwai Central China Cardiovascular Hospital and Central China Branch of National Center for Cardiovascular Diseases, Zhengzhou, China; 7 The First Affiliated Hospital, Zhejiang University School of Medicine, Hangzhou, China

**Keywords:** Atherosclerosis, Methylation

## Abstract

Atherosclerosis is the underlying cause of cardiovascular disease. Recent studies have shown that *N*^6^-methyladenosine (m^6^A) modification in macrophages is associated with atherosclerosis progression. However, there is a lack of systemic research on the role of m^6^A modification in macrophage differentiation and activation during atherosclerosis. Here we conducted multiomics analysis (MeRIP-seq and RNA-seq) of macrophages during their differentiation and activation to elucidate the regulatory network of the m^6^A spectrum at different stages. Western blot, quantitative PCR (qPCR), RNA-seq and RNA immunoprecipitation (RIP)–qPCR results demonstrated that m^6^A modification modulates KDM6B expression during macrophage activation. Through co-immunoprecipitation, RIP‒qPCR and genetic perturbation experiments, we revealed that Mettl3/Rbm15 regulates the stability of *Kdm6b* mRNA and that Kdm6b is required for interacting with and demethylating Jak1 to induce its phosphorylation-mediated macrophage activation. Next, through the analysis of single-cell RNA-seq data and coculture experiments, we revealed that Kdm6b-mediated macrophage activation promoted cytotoxic T lymphocyte cytotoxicity following atherosclerosis progression. Moreover, the systemic use of STM2457, a METTL3 inhibitor, revealed the importance of m^6^A modification in immune cell infiltration and plaque activation. Finally, we utilized macrophage-specific *Kdm6b*-knockout mice to determine whether Kdm6b facilitates macrophage and cytotoxic T lymphocyte activation and atherosclerosis. Our findings revealed that m^6^A modification plays a pivotal role in the upregulation of Kdm6b in response to IFN-γ stimulation, which is essential for the phosphorylation of Stat1-induced macrophage activation-mediated atherosclerosis development.

## Abstract (web summary)

In this study, we conduct meRIP-seq analysis on macrophages during their differentiation and activation to elucidate the landscape of the m^6^A spectrum across different stages. Combined analysis of RNA-seq and meRIP-seq revealed that Kdm6b interacts with and demethylates Jak1, promoting its phosphorylation-mediated activation of macrophages. Activated macrophages interact with T cells to promote atherosclerosis progression.

## m6A modification drives macrophage activation in atherosclerosis

Atherosclerosis, a major cause of heart disease and stroke, involves inflammation and immune responses. Researchers have found that a protein called KDM6B plays a role in this process. The study explored how KDM6B affects immune cells called macrophages during atherosclerosis. Researchers used mice to study macrophages, which are immune cells that can become activated and contribute to inflammation. They focused on a chemical modification called m^6^A on RNA, which affects how genes are expressed. They used techniques such as meRIP-seq to analyze these modifications. They found that m^6^A modifications are important for macrophage activation and that KDM6B helps regulate this process. When KDM6B was inhibited, it reduced inflammation and slowed down atherosclerosis progression in mice. The study concludes that targeting KDM6B could be a potential strategy to treat atherosclerosis by reducing inflammation.

This summary was initially drafted using artificial intelligence, then revised and fact-checked by the author.

## Graphical abstract

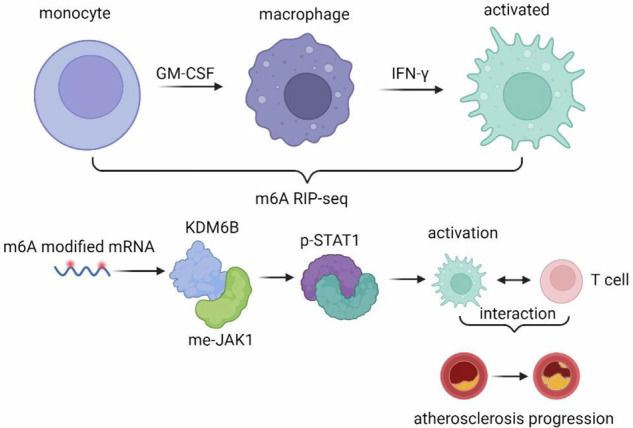

## Introduction

Atherosclerosis underlies cardiovascular diseases, including coronary heart disease and stroke, and their associated complications, making it the leading cause of death worldwide^1^. Recent studies have highlighted the critical role of immune-inflammatory responses, which are characterized by the infiltration of monocytes, macrophages and T cells, in accelerating atherosclerosis progression^1^. Upon engulfing oxidized lipoproteins via scavenger receptors, peripheral blood monocyte-derived macrophages become activated and secrete numerous proinflammatory factors, recruiting additional immune cells, including T cells, and thereby intensifying inflammation within atherosclerotic plaques^2^.

As a histone demethylase, KDM6B (also known as JMJD3) specifically targets the trimethylation of lysine 27 on histone H3 (H3K27me3), a repressive epigenetic marker associated with gene silencing^3^. By removing H3K27me3, KDM6B activates the transcription of genes involved in critical biological processes, including cell differentiation, immune responses and development^4^. Unlike its homolog UTX (KDM6A), KDM6B is activated by stimuli such as inflammation, infection or cellular stress, positioning it as a dynamic regulator of gene expression in response to environmental cues^5,6^. In macrophages, Kdm6b induces NF-κB-mediated inflammatory gene transcription, which is critical for amplifying local inflammation in abdominal aortic aneurysms^7^. Experimental knockout of Kdm6b in foam cells delays profibrotic gene transcription, suggesting that Kdm6b is closely connected with atherosclerosis progression^8^. Other studies also revealed that downregulation of Kdm6b shifts M1 polarization toward the M2 phenotype (characterized by CD163⁺ markers) of macrophages^9^. However, whether KDM6B is manipulated during macrophage activation and whether m^6^A modification regulates KDM6B expression in macrophages during atherosclerosis development are unknown.

*N*^6^-methyladenosine (m^6^A) represents the most prevalent epitranscriptional RNA modification in eukaryotic cells and affects various types of RNA, including mRNAs, tRNAs, noncoding RNAs and microRNAs^10^. m^6^A modifications on mRNAs are precisely regulated by a set of ‘writers’, such as METTL3, METTL14 and RBM15, and ‘erasers’, such as FTO and ALKBH5^11^. Research has indicated that the methyltransferase Mettl14 enhances the NF-κB/IL-6 signaling pathway, driving an inflammatory response by modulating *Myd88* mRNA stability in macrophages during atherosclerosis^12^. Furthermore, the RNA-binding protein Matr3 can regulate the depolymerization of the m^6^A modification complex, which includes Mettl3 and Mettl14, thereby mitigating the mitogen-activated protein kinase (MAPK)-mediated proinflammatory response in atherosclerotic macrophages^13^. Despite extensive research on the role of m^6^A modification and its associated factors in atherosclerotic macrophages^14–17^, a systematic investigation into m^6^A modification in monocyte-derived macrophages and its influence on their activation during atherosclerosis progression is still lacking. In this study, our team conducted methylated RNA immunoprecipitation (RIP) sequencing (meRIP-seq) on granulocyte–macrophage colony-stimulating factor (GM-CSF)-treated monocytes and IFN-γ-stimulated macrophages to provide a comprehensive understanding of the dynamic role of m^6^A modification in macrophage activation and its contribution to atherosclerosis.

## Materials and methods

### Cell culture

Primary bone marrow-derived monocytes (BMDMs) were isolated from wild-type 6- to 8-week-old C57BL/6 mice. Monocytes were cultured in complete medium supplemented with 10% fetal bovine serum, 100 μg/ml streptomycin and 100 U/ml penicillin in 5% CO_2_ at 37 °C in a humidified incubator. For macrophage differentiation, GM-CSF (20 ng/ml, cat. no. 315-03-20UG, PeproTech) was added daily for a period of 5 days. For macrophage activation, interferon-gamma (IFN-γ) (20 μg/ml, cat. no. 315-05-100UG, PeproTech) was added for 12 h. Following these distinct stimuli, the cells were collected for further experiments.

### Western blotting and co-IP

The cells were collected and lysed on ice. The protein concentration was determined using a BCA protein assay kit (cat. no. P0012, Beyotime), and 5 μg of protein was loaded onto 5–20% gradient sodium dodecyl sulfate–polyacrylamide gel. After transfer onto polyvinylidene fluoride membranes, the blots were incubated with primary antibodies at 4 °C overnight, followed by incubation with specific secondary antibodies (1:5000; goat anti-rabbit IgG H&L, cat. no. ab6721 and cat. no. ab205719, Abcam) for 1 h at room temperature. The immunoreactive signals were visualized using an enhanced chemiluminescence detection kit (cat. no. 32106, Pierce).

For co-immunoprecipitation (co-IP) assays, 1 × 10^7^ macrophages were lysed with cell lysis buffer (cat. no. P0013C, Beyotime) on ice for 30 min. After preclearing by incubation with 5 μl of magnetic beads (cat. no. LSKMGAG02, MERCK) for 1 h, the cell lysates were incubated with anti-Jak1 antibody with rotation at 4 °C overnight. After incubation with 10 μl of magnetic beads for another 1 h at 4 °C with rotation, the magnetic beads were washed three times with cold cell lysis buffer, after which elution was performed using protein lysis buffer. Then, the magnetic beads were mixed with 10 μl of 5× loading buffer (cat. no. P0286, Beyotime) and boiled for 10 min for subsequent western blotting. The primary antibodies used were as follows: mouse anti-STAT1 (1:1000, cat. no. ab239360, Abcam), rabbit anti-phosphorylated STAT1 (p-STAT1, 1:1000, cat. no. 9167, CST), rabbit anti-KDM6B (1:1000, cat. no. 3457, CST), rabbit anti-Jak1 (1:1000, cat. no. 50996, CST), rabbit anti-p-Jak1 (1:1000, cat. no. 74129, CST), rabbit anti-Flag (1:1000, cat. no. 14793, CST), rabbit anti-Myc (1:1000, cat. no. 2276, CST), rabbit anti-panmethylation (1:1000, cat. no. 7315, Abcam), rabbit anti-NSD3 (1:1000, cat. no. 300489, Abcam), rabbit anti-DOT1L (1:1000, cat. no. ab239358, Abcam), rabbit anti-H3K79me3 (1:1000, cat. no. ab208189, Abcam), rabbit anti-H3K36me3 (1:1000, cat. no. 282596, Abcam), rabbit anti-H3K27me3 (1:1000, cat. no. 6002, Abcam), rabbit anti-H3 (1:1000, cat. no. ab1791, Abcam) and rabbit anti-GAPDH (1:1000, cat. no. ab8245, Abcam).

### Reverse transcription quantitative polymerase chain reaction (RT–qPCR)

RT–qPCR was performed as briefly described: total RNA was extracted and reversed transcription was performed before qPCR was conducted using the SYBR Green mix kit (cat. no. B110031, Sangon Bio). Primers used were as follows: Kdm6b: F-5′ TGAAGAACGTCAAGTCCATTGTG-3′, R-5′ TCCCGCTGTACCTGACAGT-3′; GAPDH: F-5′ AGGTCGGTGTGAACGGATTTG-3′, R-5′ GGGGTCGTTGATGGCAACA-3′; Rbm15: F-5′ GCGAGTCCGCTGTGTGAAA-3′, R-5′ TCCCCACGAGAACTGGAGTC-3′.

### Flow cytometry

Tissue-infiltrating lymphocytes were isolated from mice, and a single-cell suspension was prepared. First, the cells were incubated with an Fc receptor blocking solution (cat. no. 553141, BD) for 15 min at 4 °C to minimize nonspecific binding. Second, the cells were stained with the respective fluorochrome-conjugated antibodies against surface antigens for 30 min at 4 °C. After being washed with phosphate-buffered saline, the cells were incubated with permeabilization buffer (1×, BD) for 30 min and then stained with intracellular antigen-conjugated antibodies for 60 min at 4 °C. Finally, after being washed with permeabilization buffer, the cells were analyzed with a BD FACSCanto II. The data were analyzed via FlowJo X software (TreeStar). The antibodies used were as follows: FVS780 (cat. no. 565388, BD), V450 anti-CD45 (cat. no. 75-0451-U100, clone 30-F11, Tonbo), FITC anti-CD107a (cat. no. 121605, clone 1D4B, BioLegend), FITC anti-CD11b (cat. no. 101205, clone M1/70, BioLegend), PE anti-F4/80 (cat. no. 111603, clone W20065B, BioLegend), APC anti-CD20 (cat. no. 161403, clone QA18A73, BioLegend), BV510 anti-CD3e (cat. no. 100233, clone 17A2, BioLegend), Percp5.5 anti-CD8a (cat. no. 65-0081-U100, clone 53-6.7, Tonbo), PE-cy7 anti-PD1 (cat. no. 109110, clone RMP1-30, BioLegend), PE-cy7 anti-GZMB (cat. no. 25-8898-82, clone NGZB, Invitrogen), V450 anti-CD86 (cat. no. 560377, clone FUN-1, BD), V510 anti-CD80 (cat. no. 104741, clone 16-10A1, BioLegend), PE-cy7 anti-Ly6C (cat. no. 128017, clone HK1.4, BioLegend).

Gating strategies were as follows: (1) for macrophages, gating was based on FSC-A versus SSC-A, FSC-A versus FSC-H, FVS780 versus CD45 and F4/80 versus SSC-H; (2) for cytotoxic T lymphocytes (CTLs), gating was based on FSC-A versus SSC-A, FSC-A versus FSC-H, FVS780 versus CD45 and CD3e versus CD8a.

### MeRIP-seq

RIP was conducted with a Magna RIP RNA-Binding Protein Immunoprecipitation Kit (cat. no. 17-700, Millipore), as previously described^18^. The enriched RNA and input RNA (as controls) were purified and subjected to library preparation and high-throughput sequencing by Novo Co., Ltd. using the Illumina platform. Raw reads in FASTQ format were processed using fastp (v0.19.11) to remove adapter sequences, reads containing more than 10% unknown bases (poly-N) and low-quality reads (defined as those with >50% of bases having a Phred quality score ≤20). Quality metrics, including the Q20, Q30 and GC contents, were calculated. Clean reads were retained for downstream analyses. The mouse reference genome (GRCm38/mm10) and Gene Transfer Format (GTF) annotation files were downloaded from the Ensembl genome database. Reference index files were constructed using BWA (v0.7.12), and clean reads were aligned to the genome using the BWA-MEM algorithm with default parameters. Only uniquely mapped reads were retained for peak calling. m⁶A peaks were identified using the exomePeak R package (v2.16.0), with the matched input RNA serving as the control. Peaks with a *q* value <0.05 were considered significantly enriched. For motif analysis of m⁶A-enriched regions, HOMER (v4.9.1) was used to identify consensus m⁶A motifs. The identified peaks were annotated to genes using the ChIPseeker. Peaks located in exonic regions were assigned to their corresponding genes, which were considered m⁶A peak-associated genes for downstream functional enrichment analysis. Differential m⁶A peak analysis was performed using the exomePeak R package (v2.16.0). Differentially methylated peaks between experimental groups were identified with a *P* value <0.05 and a fold change >1 as significance thresholds. Peaks showing statistically significant changes in m⁶A enrichment were defined as differentially methylated regions.

### RNA sequencing (RNA-seq)

The macrophages were pretreated with GSKJ1 (cat. no. HY-15648, MCE) before IFN-γ stimulation. Total RNA was extracted using TRIzol reagent (cat. no. 15596026CN, Invitrogen). The RNA samples were sent to Novo Co., Ltd. for sequencing using the Illumina Genome Analyser II platform (Illumina). Raw sequencing data in FASTQ format were first subjected to quality control. Adapter-containing reads, reads with more than 10% unknown nucleotides (N) and low-quality reads (defined as those in which more than 50% of the bases had Phred scores ≤20) were removed using Trimmomatic (v0.39). Quality metrics, including Q20, Q30 and GC content, were calculated, and the resulting clean reads were used for all downstream analyses. For alignment, clean reads were aligned to the mouse reference genome (GRCm38/mm10) using HISAT2 (v2.0.5). The aligned reads of each sample were assembled into transcripts using StringTie (v1.3.3b) in reference-guided mode. Gene expression quantification was performed using featureCounts (v1.5.0-p3) to calculate the number of reads mapped to each gene. Gene expression levels were normalized as fragments per kilobase of transcript per million mapped reads (FPKM) values. Differential expression analysis between two experimental conditions (with biological replicates) was performed via DESeq2 (v1.20.0). A negative binomial distribution model was applied to estimate dispersion and test for significance. The resulting *P* values were adjusted for multiple testing using the Benjamini‒Hochberg method. Genes with adjusted *P* values <0.05 were considered differentially expressed. Significant genes were defined on the basis of a corrected *P* value <0.05 and an absolute fold change ≥2.

### MeRIP–qPCR

The meRIP assay was performed as described previously with slightly modifications^19^. In brief, 10–20 μg RNA was fragmented into 200–300-nt fragments via a 15-min incubation at 70 °C in fragmentation buffer (10 mM ZnCl_2_ and 10 mM Tris–HCl pH 7.0). For exogenous *Kdm6b* methylation analysis, RNA fragmentation was omitted to preserve full-length transcripts. Thereafter, the fragmented RNA was incubated with 2–3 μg m^6^A antibody (cat. no. 202003, Synaptic Systems) at 4 °C overnight, and then the complex was incubated with protein G beads (Invitrogen) overnight at 4 °C. After washing with meRIP lysis buffer (150 mM NaCl, 0.1% NP-40 and 10 mM Tris–HCl), the bound RNAs were recovered by proteinase K digestion, phenol–chloroform extraction and ethanol preparation. One-tenth of the fragmented RNA was used as an input control. The input or bound RNA was subjected to reverse transcription and subsequent qPCR. Relative enrichment was calculated as fold change relative to IgG controls after normalization to input RNA. Primers used were as follows: Kdm6b: F-5′ AATGGAAGAGCGGCGCGCTGCG-3′, R-5′ TCTGTACAGAACTGTAGCAGGACC-3′.

### Immunofluorescence (IF) staining

IF staining was performed as previously described^20^. Primary antibodies, including rabbit anti-JAK1 (1:200, cat. no. ZRB1915, MERCK) and rabbit anti-KDM6B (1:200, cat. no. PA5-22974, Invitrogen), were used. The nuclei were stained with DAPI (cat. no. ab104139, Abcam) and examined with a fluorescence microscope (Olympus).

### Oil Red O staining

The macrophages were subjected to pretreatment with GSKJ1 before oxidized low-density lipoprotein (oxLDL) (40 μg/ml, cat. no. YB-002, YiYuan) treatment for 24 h. After they were washed twice with phosphate-buffered saline, the macrophages were stained with Oil Red O working solution (cat. no. D027-1-2, JianCheng) for 10 min and compound staining solution for 10 min. All steps of the staining procedure were conducted at room temperature.

### MiloR analysis

MiloR analysis, which is primarily used to identify phenotype-related perturbations at the single-cell level, was performed as previously described (https://github.com/MarioniLab/miloR). In brief, after the samples were refined to capture continuous trajectories, a count matrix from single-cell (sc)RNA-seq was used to perform differential abundance testing using the *k*-nearest neighbor graph method. The main parameters used are as follows: (1) *k* = 30 and *d* = 20 for the buildGraph function; (2) prop = 0.2, *k* = 30, *d* = 20 and refined = TRUE for the makeNhoods function; and (3) *d* = 20 for the calcNhoodDistance function.

### Analysis of mRNA stability

Analysis of mRNA stability was performed as described previously^21^. To determine the half-life of endogenous *Kdm6b* mRNA, actinomycin D (2 μg/ml, cat. no. SBR00013, MERCK) was added to the cell culture medium after the cells had been transfected with Mettl3 or Rbm15 small interfering RNA for 48 h. Cells were collected at the indicated time points. The total RNA was extracted by TRIzol reagent and subjected to RT–qPCR analysis.

### Animal experiments

Macrophage-specific *Kdm6b*-knockout (CKO) mice were generated via CRISPR–Cas9-based targeting and the cre-loxp (*Csf1r*-cre + *Kdm6b*-loxp) system on the *Ldlr*^*−/−*^ background by Jicui Biosciences (China). *Ldlr*^*−/−*^ mice and C57BL/6 mice were purchased from the Model Animal Research Center of Nanjing. All the mice were housed at the Ningbo University Laboratory Animal Center with controlled temperature and humidity and a 12:12 h dark‒light cycle and were provided water and mouse chow ad libitum.

Wild-type, *Ldlr*^*−/−*^ and CKO mice were fed with a high-fat diet (Research diests, D12108c) for 16 weeks to induce atherosclerosis. STM2457 (cat. no. HY-134836, MCE) was administered by intraperitoneal injection at a daily dose of 10 mg/kg. The mice used in the in vitro experiments were euthanized humanely using CO_2_ gas. The aorta was meticulously dissected from the aortic arch to the iliac bifurcation, opened longitudinally, and stained with Oil Red O as described above. The hearts were then embedded in OCT compound (Tissue-Tek, Sakura), rapidly frozen, and from the aortic sinus, consecutive 5-μm-thick cryosections were meticulously cut. These sections underwent sequential staining with Oil Red O, Masson’s trichrome staining and hematoxylin–eosin staining as we described previously^22^. All acquired images were rigorously analyzed by a seasoned investigator who was blinded to the specific mouse genotypes; the representative micrographs were quantified for lesion area utilizing ImageJ software. The lesion area in the en face preparations was articulated as a percentage of the overall aortic surface area, consistent with prior methodology. Echocardiograms were obtained using a Vevo 3100 Ultrasound System as we described previously^23^. Mouse biochemical indicators in serum were analyzed using ADVIA 2120i system. All experimental procedures were approved by the Animal Care Ethics Committee of Ningbo University (application number 13720) and conformed to the guidelines from Directive 2010/63/EU of the European Parliament on the protection of animals used for scientific purposes.

### Statistical analysis

The data are presented as the means ± standard deviations. Statistical comparisons between two groups were performed using an unpaired two-tailed Student’s *t*-test, whereas comparisons among multiple groups were analyzed via one-way analysis of variance. A *P* value of <0.05 was considered statistically significant. All the experiments were repeated at least three times. Statistical analyses were performed with GraphPad Prism software.

## Results

### Dynamic changes in the m^6^A spectrum during macrophage differentiation

**Fig. 1:**
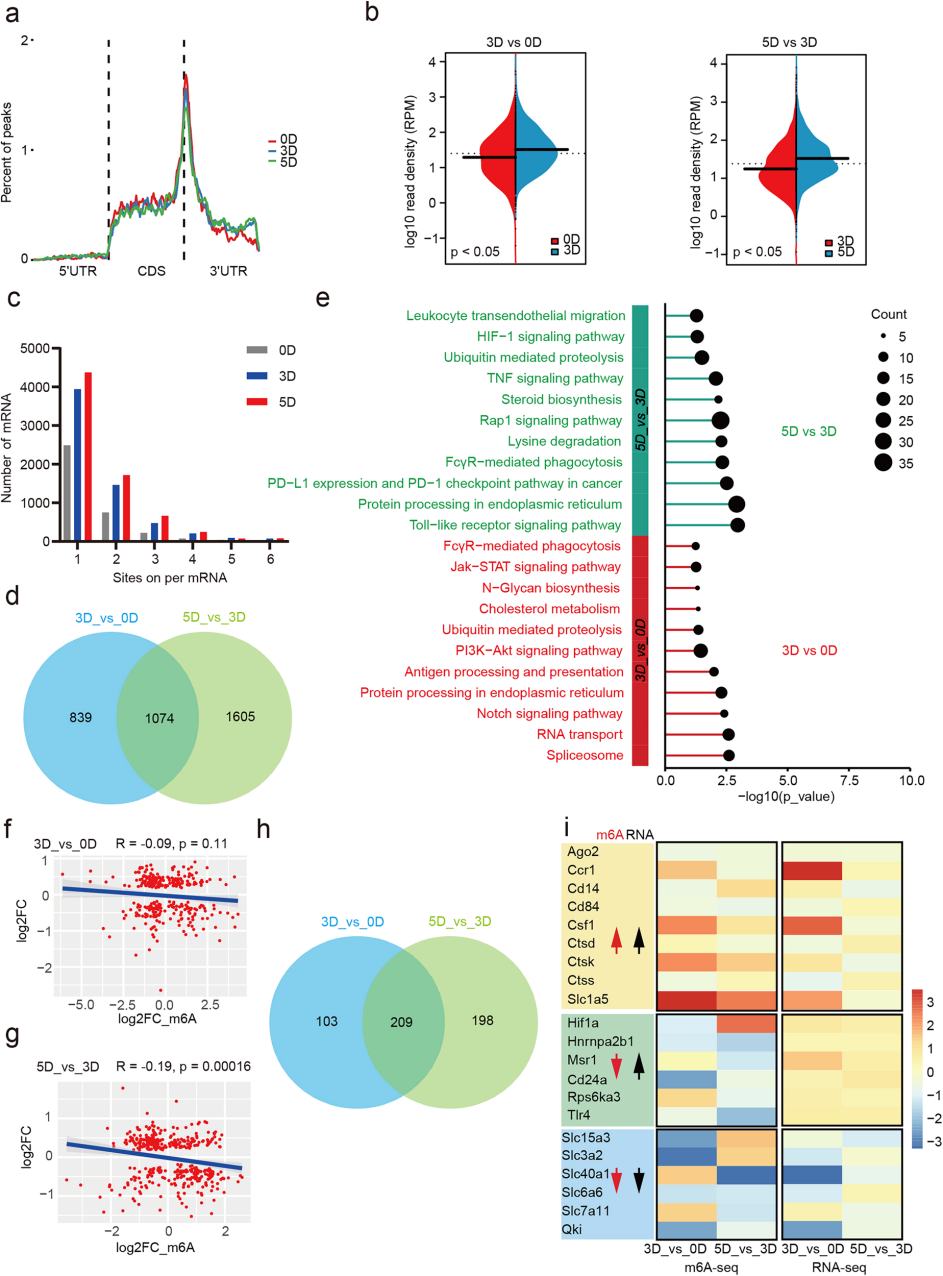
m^6^A modification landscape in monocyte-derived macrophages. Monocytes were stimulated with GM-CSF to induce macrophage differentiation. MeRIP-seq was performed using monocytes incubated with GM-CSF for 0 days, 3 days and 5 days (0D, 3D and 5D, respectively). **a**, The distribution of m^6^A peaks in gene body regions, including the 5′ UTR, CDS and 3′ UTR, among the three groups. **b**, The density of differential m^6^A peaks was compared between the indicated groups. **c**, Bar plot showing the number of mRNAs containing m^6^A sites in the three groups. **d**, Venn diagram showing the number of shared genes detected between the indicated groups. **e**, KEGG pathway enrichment analysis of DEGs was compared between the indicated groups. **f**,**g**, Correlation analysis of gene expression levels and changes in m^6^A modifications in the 3D_vs_0D group (**f**) or in the 5D_vs_3D group (**g**). The *x* axis denotes the change in mRNA (RNA-seq); the *y* axis denotes the change in m^6^A peaks (RIP-seq). **h**, Venn diagram showing the number of shared genes detected from RNA-seq and RIP-seq between the indicated groups. **i**, Heatmap showing the differences in expression and m^6^A modifications between the 3D and 0D groups or between the 5D and 3D groups.

We initially cultured BMDMs with GM-CSF to induce their differentiation into macrophages (Supplementary Fig. 1a) and performed meRIP-seq at days (D) 0, 3 and 5. As expected, the m^6^A modification peaks were predominantly found surrounding the stop codon in the mRNA transcripts (Fig. 1a and Supplementary Fig. 1b). Interestingly, m^6^A modification-related enzymes were almost upregulated following GM-CSF stimulation (Supplementary Fig. 1c). Moreover, both the density of m^6^A modifications and the number of m^6^A-containing mRNAs increased following GM-CSF treatment (Fig. 1b–d). Inhibition of m^6^A modification suppressed macrophage differentiation (Supplementary Fig. 1d), which suggests that m^6^A modification is crucial for macrophage differentiation. Kyoto Encyclopedia of Genes and Genomes (KEGG) enrichment analysis of the differentially expressed genes (DEGs) between the 5D group and the 3D group or between the 3D group and the 0D group revealed that various pathways were regulated, including those related to immunity (antigen processing and presentation, FcγR-mediated phagocytosis and Toll-like receptor signaling), inflammation (JAK–STAT signaling and TNF signaling) and molecular processing (ubiquitin-mediated proteolysis, steroid biosynthesis, lysine degradation and N-glycan biosynthesis) (Fig. 1e). These findings indicate that m^6^A methylation may participate in the remodeling of immunological activities. A combined analysis of RNA-seq and meRIP-seq revealed a negative correlation between mRNA expression levels and m^6^A modification, which was particularly pronounced in the group cultured for 5 days (Fig. 1f–h). Notably, the mRNAs of *Ccr1*, *Csf1*, *Hif1a* and *Tlr4* were significantly induced after 5 days of stimulation; however, the changes in m^6^A modifications within these mRNAs were markedly different (Fig. 1i), suggesting that different regulatory mechanisms are involved. Collectively, these results indicate that m^6^A modifications are involved in the process of macrophage differentiation.

### Proinflammatory pathways and epigenetic enzymes are specifically manipulated by m^6^A modification during macrophage activation

**Fig. 2:**
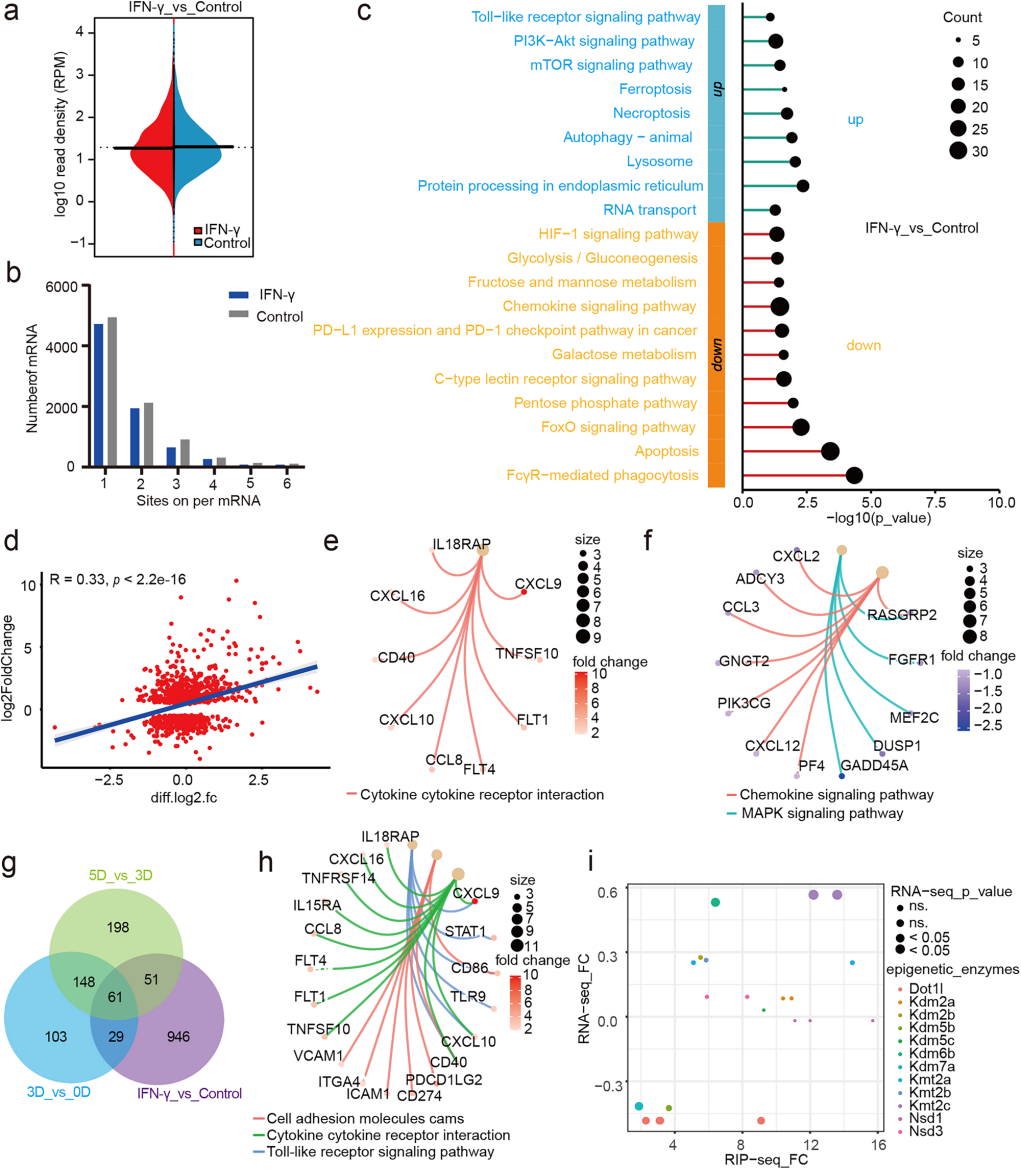
m^6^A modification landscape in IFN-γ-activated macrophages. Macrophages were stimulated with IFN-γ to induce macrophage activation. MeRIP-seq was performed using macrophages incubated with or without IFN-γ (control and IFN-γ groups). **a**, The density of differential m^6^A peaks was compared between the indicated groups. **b**, Bar plot showing the number of mRNAs containing m^6^A sites in the two groups. **c**, KEGG pathway enrichment analysis of DEGs between the indicated groups. **d**, Correlation analysis of differential genes and m^6^A modification changes between the IFN-γ group and the control group. The *x* axis denotes the change of mRNA (RNA-seq); the *y* axis denotes the change of m^6^A peaks (RIP-seq). **e**, KEGG pathway enrichment analysis of genes in the first quadrant of **d**. **f**, KEGG pathway enrichment analysis of genes in the third quadrant of **d**. **g**, Venn diagram showing the number of overlapping genes among the indicated groups. **h**, KEGG pathway enrichment analysis of genes detected only in IFN-γ**-**activated macrophages and not in GM-CSF-treated macrophages. **i**, Scatter plot showing the fold changes in the mRNA and m^6^A modification levels of the indicated epigenetic enzyme mRNAs.

To investigate the alterations in the m^6^A modification landscape during macrophage activation, we conducted meRIP-seq on macrophages after stimulation with IFN-γ. Our findings revealed that, in contrast to the differentiation phase, macrophage activation did not significantly alter the overall density of m^6^A modifications (Fig. 2a,b and Supplementary Fig. 1e,f). However, m^6^A modification is also necessary for IFN-γ-induced macrophage activation (Supplementary Fig. 1g,h). KEGG enrichment analysis of DEGs between the IFN-γ and control groups revealed that the PI3K–Akt signaling and Toll-like receptor signaling pathways were upregulated, whereas the PD-1/PD-L1 checkpoint pathway and FcγR-mediated phagocytosis were downregulated (Fig. 2c). A correlation analysis integrating RNA-seq and meRIP-seq data indicated that m^6^A modification is positively correlated with mRNA expression levels during macrophage activation (Fig. 2d). Notably, cytokine‒cytokine receptor interactions, chemokine signaling and MAPK signaling pathways were consistently regulated at both the mRNA and m^6^A modification levels (Fig. 2e,f). However, IFN-γ stimulation also induced a specific cluster of genes distinct from those expressed by GM-CSF (Fig. 2g). KEGG enrichment analysis of these genes that were regulated only in the IFN-γ group revealed that the expression of macrophage maturity-related genes, such as *Stat1*, *Cd86*, *Cxcl10* and *Cd274*, was specifically modulated during the activation process (Fig. 2h). Intriguingly, a substantial group of epigenetic modification enzymes, including *Dot1l*, *Kdm6B*, *Kdm5b* and *Kmt2c*, also appeared to be regulated in only the IFN-γ group (Fig. 2i). Because epigenetic modification enzymes regulate gene transcription at the initial stage, we focused on this cluster of genes. To determine the basal expression levels and specificity of these enzymes in macrophages, their expression was also examined via scRNA-seq data from a previous study^24^. After rigorous integration and reduced dimension analysis, we obtained 16 clusters of immune cells, including macrophages and T cells (Supplementary Fig. 2a–c). As atherosclerosis progresses, the proportions of macrophages and T cells increase (Supplementary Fig. 2d). Unexpectedly, with the exception of Kdm6b and Nsd3, the majority of these enzymes were scarcely expressed in immune cells derived from atherosclerotic plaques, with Kdm6b expression observed predominantly in myeloid cells (Supplementary Fig. 2e). Indeed, IFN-γ stimulation markedly enhanced m^6^A modification of Kdm6b mRNA and resulted in the upregulation of Kdm6b (Supplementary Fig. 3a–d). Interestingly, the induction of H3K27me3, H3K36me3 and H3K79me3 modifications did not markedly change after IFN-γ stimulation (Supplementary Fig. 3e). Overall, both proinflammatory pathways and the epigenetic enzyme Kdm6b are specifically manipulated by m^6^A modifications during macrophage activation, highlighting the complex regulatory network that governs immune responses.

### KDM6B promotes STAT1 phosphorylation in a demethylase activity-dependent manner

**Fig. 3:**
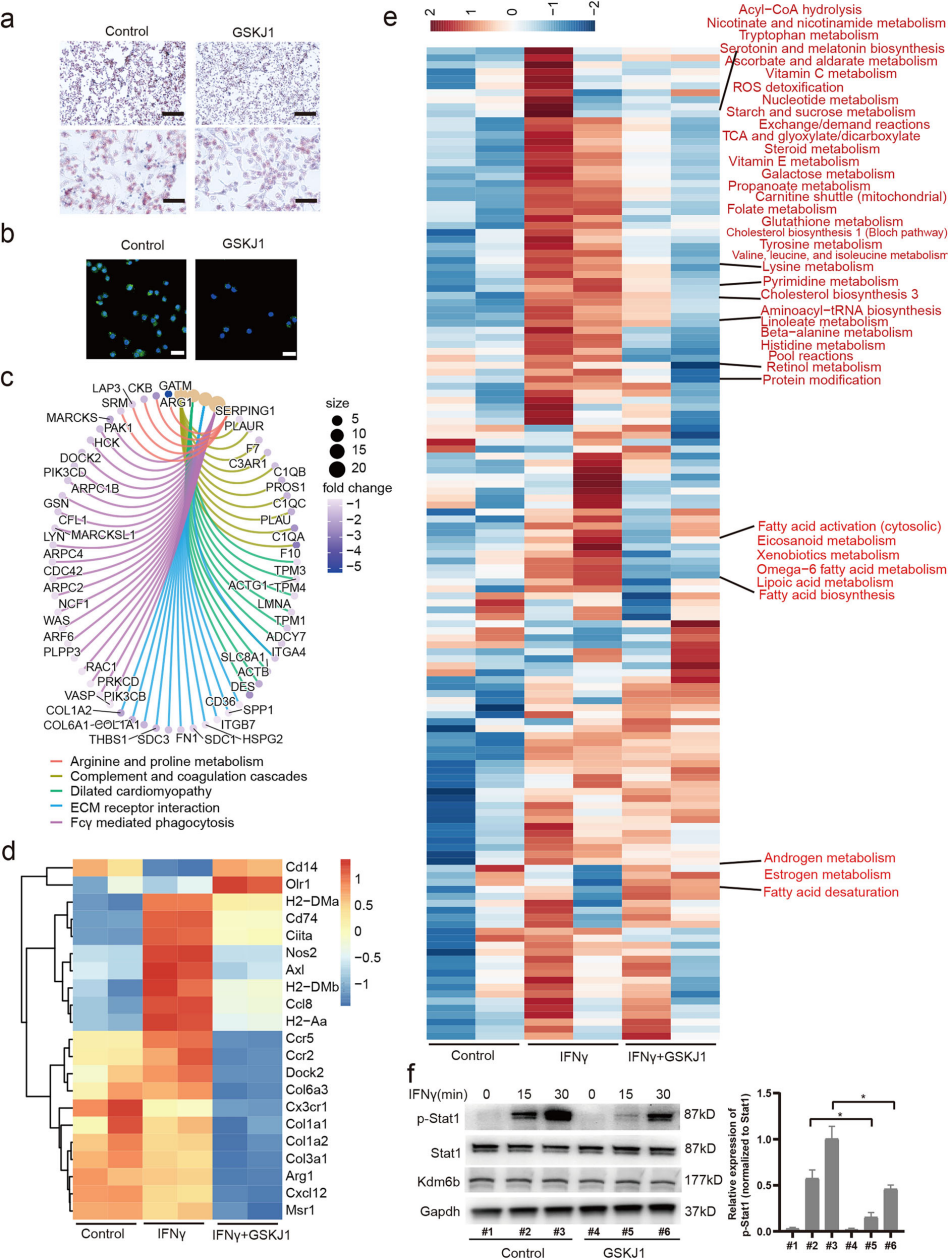
KDM6B regulates lipid uptake, ROS production and IFN-γ-induced STAT1 phosphorylation in macrophages. **a**, Macrophages were pretreated with GSKJ1 for 30 min, stimulated with IFN-γ for 12 h and subsequently incubated with oxLDL for 24 h. Lipid uptake was evaluated via Oil Red O staining. Scale bars, 200 μm (top) and 100 μm (bottom). **b**, Macrophages were pretreated with GSKJ1 for 30 min and then stimulated with IFN-γ for 12 h. Mitochondrial ROS production was detected via a ROS assay kit. Green fluorescence indicates the intensity of the ROS. Scale bar, 100 μm. The macrophages were pretreated with GSKJ1 for 30 min and then stimulated with IFN-γ for 12 h, and total RNA was subsequently collected for RNA-seq. **c**, Cnet plot showing KEGG enrichment pathways identified from DEGs between the IFN-γ-treated group and the GSKJ1 + IFN-γ-treated group. **d**, Heatmap showing DEGs among the control, IFN-γ and GSKJ1 + IFN-γ groups. **e**, Heatmap showing the enriched metabolic pathways affected by GSKJ1 treatment in macrophages, with differentially regulated pathways marked in red. **f**, Left: western blot analysis of Stat1, p-Stat1 and Kdm6b in macrophages pretreated with GSKJ1 for 30 min before they were stimulated with IFN-γ at the indicated times, with Gapdh used as an endogenous control. Right: the quantified results. *P < 0.05.

To elucidate the role of Kdm6b in macrophages, we incubated cells with GSKJ1, a specific inhibitor of Kdm6b. Our results revealed that GSKJ1 significantly reduced lipid uptake and reactive oxygen species (ROS) formation in macrophages stimulated with IFN-γ (Fig. 3a,b). We subsequently conducted RNA-seq to assess the impact of IFN-γ and GSKJ1 on gene expression during macrophage activation. As anticipated, IFN-γ stimulation enhanced pathways related to antigen processing and presentation, the cytosolic DNA-sensing pathway and PPAR signaling (Supplementary Fig. 4a–c). However, compared with IFN-γ-only treatment, GSKJ1 treatment attenuated the IFN-γ-induced upregulation of genes involved in FcγR-mediated phagocytosis, dilated cardiomyopathy and extracellular matrix receptor interaction (Fig. 3c). In addition, the expression of molecules associated with antigen presentation (*H2-DMa*, *Cd74* and *Ciita*), chemokine factors (*Ccr5*, *Ccr2*, *Cxcl12* and *Dock2*) and collagen deposition (*Col1a1*, *Col1a2* and *Col3a1*) decreased with GSKJ1 treatment, indicating a dampening effect on IFN-γ-mediated macrophage activation (Fig. 3d). Furthermore, we applied METAFlux analysis^25^ to examine metabolic gene changes during GSKJ1 incubation. Figure 3e shows that the IFN-γ-induced upregulation of acyl-CoA hydrolysis and the metabolism of various small molecules, including galactose, folate, glutathione, valine, leucine, isoleucine, lysine, pyrimidine, linoleate, β-alanine and retinol, as well as fatty acid activation and biosynthesis, were counteracted by GSKJ1. These findings suggest that the suppressive effect of GSKJ1 on IFN-γ signaling is not limited to a specific set of target genes but rather has a broad inhibitory effect. Accordingly, we investigated key events in the IFN-γ signaling cascade and discovered that either GSKJ1 or Kdm6b knockdown inhibited the phosphorylation of Stat1 (Fig. 3f and Supplementary Fig. 4d,e).

### KDM6B promotes JAK1 phosphorylation by demethylating JAK1

**Fig. 4:**
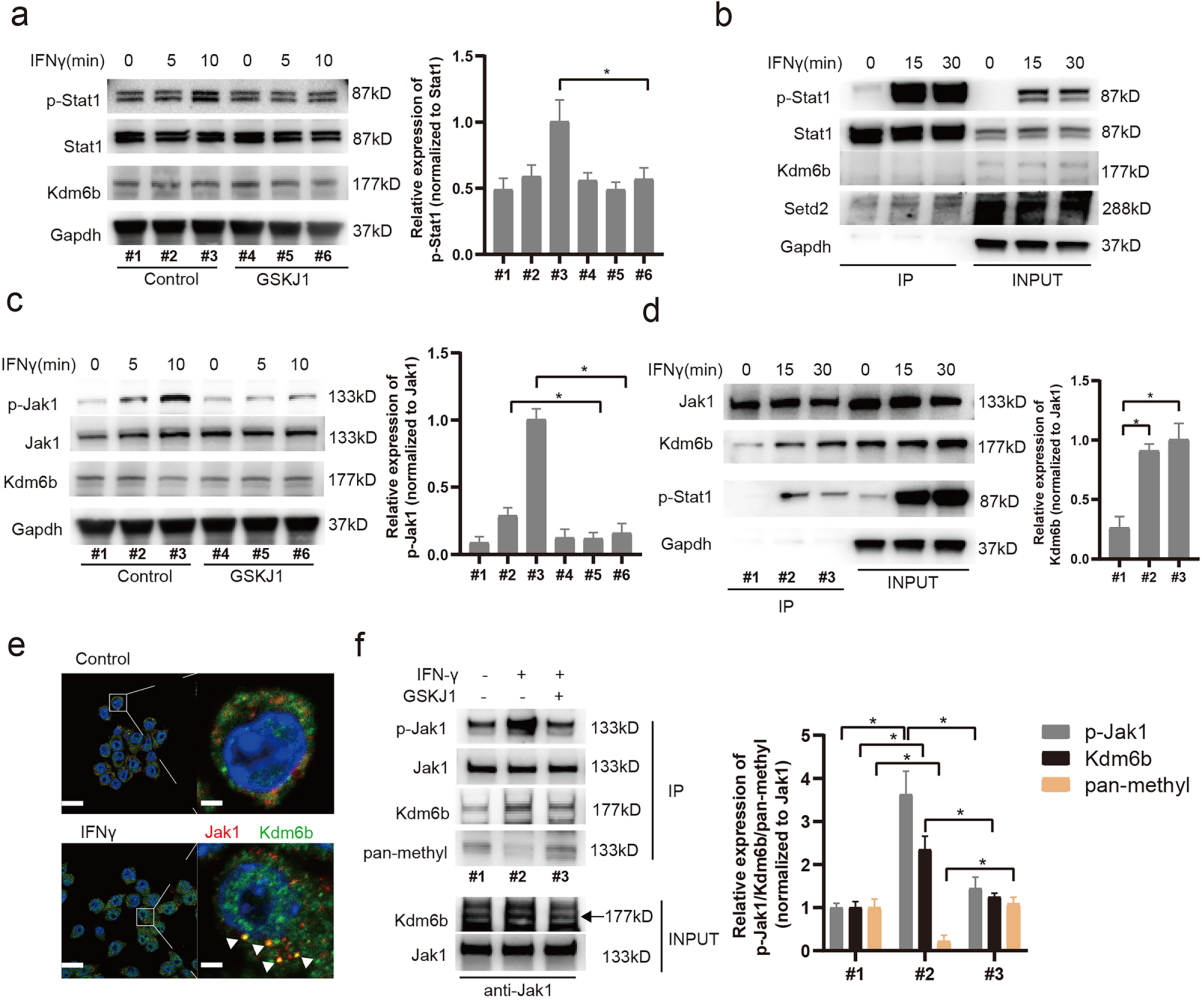
KDM6B facilitates IFN-γ signaling by interacting with Jak1 and increasing its phosphorylation. **a**, Macrophages were pretreated with GSKJ1 for 30 min and then stimulated with IFN-γ at the indicated times. Left: western blot analysis of Stat1, p-Stat1 and Kdm6b was performed, with Gapdh used as an endogenous control. Right: the quantified results. *P < 0.05. **b**, Macrophages were pretreated with GSKJ1 for 30 min and stimulated with IFN-γ at the indicated times. Co-IP assessment of the interaction between Stat1 and Kdm6b for the indicated times using an anti-Stat1 antibody. **c**, Macrophages were pretreated with GSKJ1 for 30 min and stimulated with IFN-γ at the indicated times. Left: western blot detection of Jak1, phosphorylated Jak1 (p-Jak1) and Kdm6b with Gapdh used as an endogenous control. Right: the quantified results. *P < 0.05. **d**, Macrophages were pretreated with GSKJ1 for 30 min and stimulated with IFN-γ at the indicated times. Left: co-IP analysis of the interaction between Jak1 and Kdm6b using an anti-Jak1 antibody. Right: the quantified results. *P < 0.05. **e**, Macrophages were stimulated with IFN-γ for 30 min. IF analysis of Jak1 and Kdm6b in macrophages was performed. Nuclei are visualized with DAPI. Scale bars, 50 μm (left) and 10 μm (right). **f**, Macrophages were stimulated with IFN-γ for 30 min with or without GSKJ1. Left: co-IP detection of the interaction between Jak1 and Kdm6b using an anti-Jak1 antibody. Right: the quantified results. *P < 0.05.

To confirm the role of Kdm6b in Stat1 phosphorylation, we assessed IFN-γ-induced Stat1 phosphorylation over a shorter time frame and obtained consistent results (Fig. 4a). However, because our data revealed no direct interaction between Kdm6b and Stat1 (Fig. 4b), we hypothesized that GSKJ1 might affect kinases upstream of the JAK–STAT signaling pathway. Our subsequent experiments confirmed that GSKJ1 indeed inhibits the phosphorylation of Jak1 induced by IFN-γ (Fig. 4c). Moreover, we found that IFN-γ significantly enhances the interaction between Kdm6b and Jak1 (Fig. 4d). Given that Kdm6b, an epigenetic enzyme, is primarily nuclear, its interaction with Jak1, which is predominantly cytoplasmic, raises questions about its cellular localization dynamics. To test this hypothesis, we performed colocalization staining and discovered that a subset of the Kdm6b protein indeed translocates to the cytoplasm and colocalizes with Jak1 upon IFN-γ stimulation (Fig. 4e). This observation led us to investigate whether Kdm6b demethylates Jak1 to promote its phosphorylation. To test this hypothesis, we conducted co-IP experiments in IFN-γ-stimulated macrophages. Our results revealed that the methylation of Jak1 is reduced and that the phosphorylation of Jak1 is induced after IFN-γ treatment (Fig. 4f), which suggests that this methylation is related to the phosphorylation of Jak1.

### IFN-γ induces METTL3-dependent m^6^A modifications on KDM6B mRNA

**Fig. 5:**
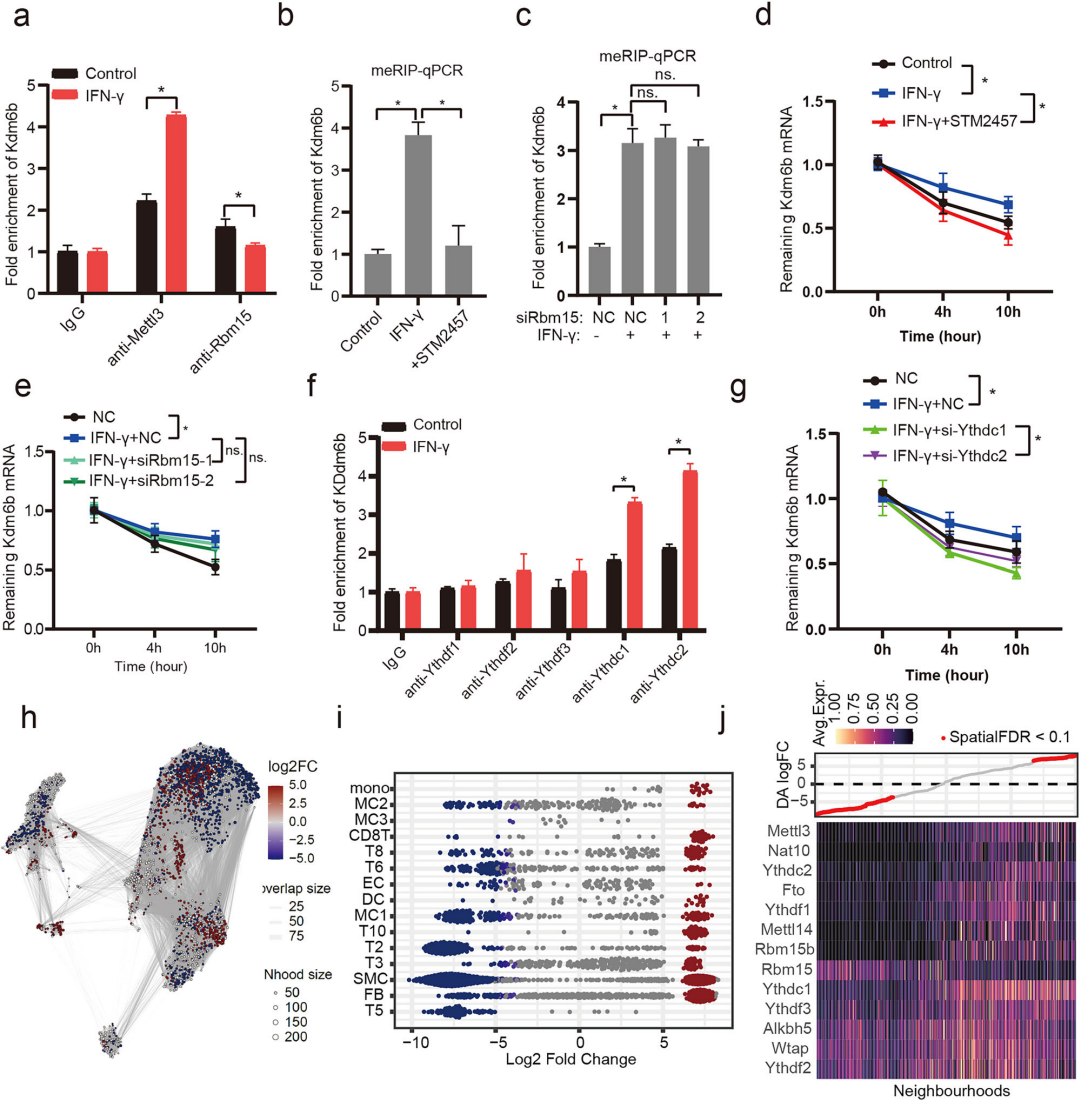
IFN-γ stimulation induces Mettl3-mediated m^6^A modifications in KDM6B mRNA. **a**, Macrophages were stimulated with IFN-γ for 12 h. An RIP assay was performed to assess the interaction between Kdm6b mRNA and the indicated antibodies in macrophages stimulated with IFN-γ. *P < 0.05. **b**, Macrophages were pretreated with STM2457 and stimulated with IFN-γ for 12 h. MeRIP-qPCR was subsequently performed. KDM6B mRNA levels were quantified by real-time qPCR. *P < 0.05. **c**, Macrophages transfected with or without si-Rbm15 were stimulated with IFN-γ for 12 h. MeRIP-qPCR was subsequently performed. KDM6B mRNA levels were quantified by real-time qPCR. *P < 0.05. **d**, Macrophages were pretreated with STM2457 and stimulated with IFN-γ for 12 h. The RNA stability of Kdm6b mRNA was detected. *P < 0.05. **e**, Macrophages transfected with or without si-Rbm15 were stimulated with IFN-γ for 12 h. The RNA stability of Kdm6b mRNA was detected. *P < 0.05. **f**, Macrophages were stimulated with IFN-γ for 12 h. An RIP assay was performed to assess the association between Kdm6b mRNA and the indicated antibodies in IFN-γ-treated macrophages. *P < 0.05. **g**, Macrophages transfected with or without si-Ythdc1 were stimulated with IFN-γ for 12 h. The RNA stability of Kdm6b mRNA was detected. *P < 0.05. **h**, Neighborhood assignment map using Louvain clustering. The dashed line encloses the region of neighborhood groups that show distinct clustering patterns of plaque scRNA-seq. **i**, Beeswarm plot showing the distribution of log fold changes in neighborhoods containing cells from various cell subsets. Neighborhoods with differential abundance at a false discovery rate of 10% are highlighted in color. **j**, Heatmap representation of DEGs among differentially abundant neighborhoods in myeloid cell subsets isolated from atherosclerotic plaques in mice.

To determine which m^6^A writers are involved in the modification of Kdm6b mRNA induced by IFN-γ, we conducted RIP-qPCR. As shown in Fig. 5a, both Mettl3 and Rbm15 interact with Kdm6b mRNA in macrophages under steady-state conditions. However, upon IFN-γ stimulation, there is an increase in the association of Mettl3 with Kdm6b mRNA and a corresponding decrease in the association of Rbm15. Inhibition of METTL3 but not knockdown of Rbm15 eliminated the IFN-γ stimulation-induced m^6^A modifications of Kdm6b mRNA (Fig. 5b,c and Supplementary Fig. 5a) and reduced its stability (Fig. 5d,e). Furthermore, to explore the downstream effects of m^6^A modification on Kdm6b mRNA, we performed RIP‒qPCR using antibodies against YTH domain-containing proteins (including Ythdf1/2/3 and Ythdc1/2) and Igf2bp1/2/3 proteins (Fig. 5f and Supplementary Fig. 5b). In conjunction with mRNA degradation assays, we discovered that Ythdc1/2 promotes the stability of Kdm6b mRNA in response to IFN-γ stimulation (Fig. 5g). Indeed, both the mRNA and protein levels of Kdm6b were reduced following m^6^A modification inhibition or Ythdc1 knockdown (Supplementary Fig. 5c,d). Given that these findings were based on in vitro cellular experiments, we questioned the relevance of m^6^A-mediated Kdm6b regulation in the context of atherosclerosis in vivo. We initially conducted miloR analysis^26^ across various time points, including the control, 4-week (w), 8w, 12w, 16w and 26w time points. We identified a significant phenotypic switch, primarily between the 16w/12w groups and the control/4w groups (Fig. 5h,i). We subsequently assessed the transcriptional changes in myeloid cells, which include monocytes and macrophages. As anticipated, we observed an increase in the expression of Mettl3 and Ythdc1/2, whereas the expression of Rbm15 decreased as pseudotime progressed (Fig. 5j).

### KDM6B-dependent macrophage activation is necessary for CTL cytotoxicity

**Fig. 6:**
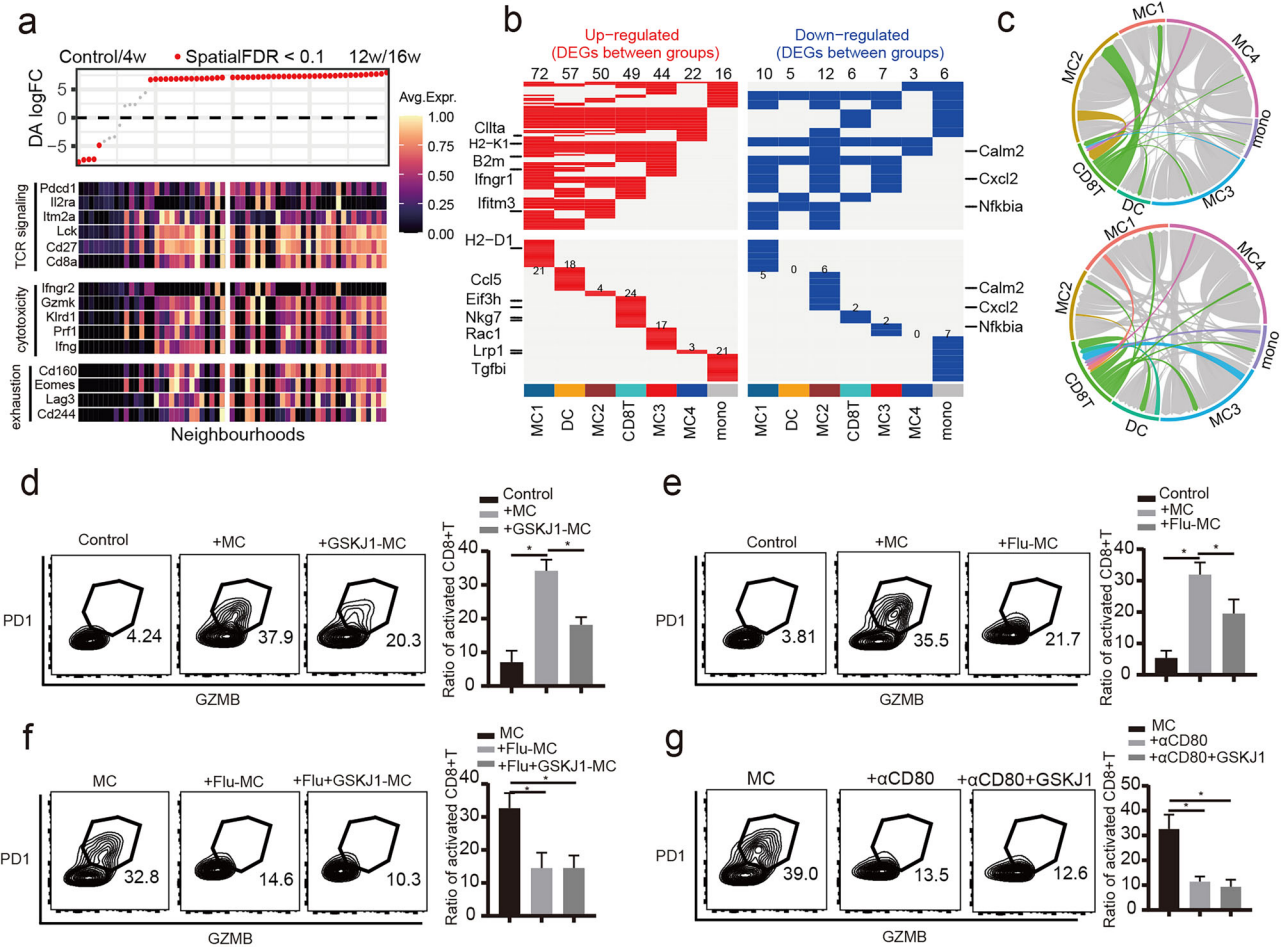
Macrophages promote CTL activation through direct interactions. **a**, Heatmap showing the DEGs among differentially abundant neighborhoods in T subsets isolated from atherosclerotic plaques in mice. **b**, Heatmap showing the DEGs in immune cell subsets isolated from atherosclerotic plaques in mice. **c**, Chord plot depicting the interactions among immune cell subsets as analyzed by the ComPath method. **d**, Macrophages were pretreated with GSKJ1 for 30 min and stimulated with IFN-γ for 12 h before being cocultured with CTLs. Flow cytometry analysis of the activated CTL ratio (PD-1^+^GZMB^+^ subset). *P < 0.05. **e**, Macrophages were pretreated with Flu (a STAT1 phosphorylation inhibitor) for 30 min and stimulated with IFN-γ for 12 h before being cocultured with CTLs. Flow cytometry measurement of the activated CTL ratio. *P < 0.05. **f**, Macrophages were pretreated with both Flu and GSKJ1 for 30 min and stimulated with IFN-γ for 12 h before being cocultured with CTLs. Flow cytometry measurement of the activated CTL ratio. *P < 0.05. **g**, Macrophages were pretreated with IFN-γ for 6 h, followed by αCD80 and GSKJ1 treatment for 6 h before being cocultured with CTLs. Flow cytometry detection of the activated CTL ratio. *P < 0.05.

While CTLs are known to promote atherosclerosis^27^, we investigated the transcriptional changes in CTLs associated with phenotypic transitions. As anticipated, we observed an upregulation of T cell receptor signaling molecules (*Pdcd1*, *Il2ra*, *Itm2a*, *Lck*, *Cd27* and *Cd8a*), cytotoxicity-related molecules (*Ifngr2*, *Gzmk*, *Klrd1*, *Prf1* and *Ifng*) and exhaustion markers (*Cd160*, *Eomes*, *Lag3* and *Cd244*), which suggests continuous antigen stimulation of CTLs within the plaque during progression (Fig. 6a). Differential gene expression analysis revealed that the antigen-presenting machinery, including *Ciita*, *H2-K1*, *B2m* and *H2-D1*, was upregulated in a macrophage subset referred to as MC1 (Fig. 6b). Notably, the expression of Ifngr1, the ligand for IFN-γ secreted by CTLs, was also increased (Fig. 6b), which suggests a potential regulatory role of CTLs in this macrophage subset. Connectome analysis^28^ supported our hypothesis by revealing an increased association between MC1 macrophages and CTLs during the progression of atherosclerosis (Fig. 6c). To explore the role of Kdm6b–IFN-γ signaling in this interaction, we conducted in vitro coculture experiments. We found that IFN-γ-activated macrophages significantly enhanced the cytotoxicity of CTLs through direct coculture with CTLs; however, this effect was reversed by the inhibition of Kdm6b or the suppression of Stat1 phosphorylation (Fig. 6d,e and Supplementary Fig. 6a,b). Furthermore, the ability of Kdm6b to promote macrophage-induced CTL cytotoxicity was dependent on both Stat1 phosphorylation and CD80 signaling (Fig. 6f,g). These results suggest that the interaction between macrophages and CTLs is crucial for the development of atherosclerosis.

### Macrophage-specific KDM6B deficiency delays atherosclerosis

**Fig. 7:**
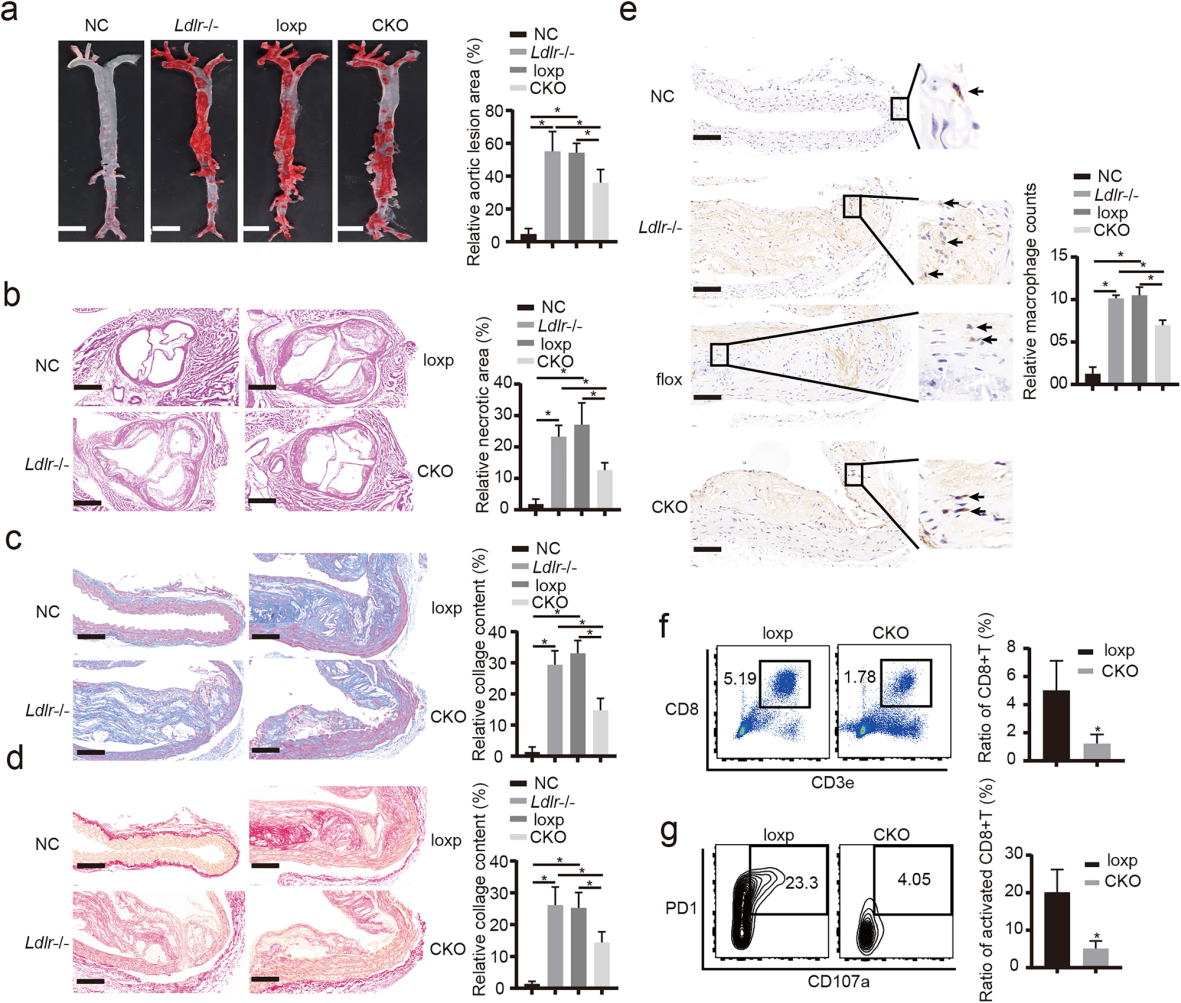
Macrophage-specific Kdm6b deficiency delays atherosclerosis development. **a**, Representative images and quantification of Oil Red O staining of aorta en face lesions isolated from the NC *(Ldlr*^−/−^ mice fed with a chow diet), *Ldlr*^−/−^, flox and CKO groups (*n* = 6 per group). *P < 0.05. **b**, Hematoxylin and eosin staining images and quantification of the necrotic core area in aortic sections from the NC, *Ldlr*^−/−^, flox and CKO groups (*n* = 6 per group). *P < 0.05. **c**–**e**, Histological analysis with Masson’s trichrome staining (**c**), Sirius red staining (**d**) and MOMA2 (macrophage/monocyte monoclonal antibody) staining (antigen: mouse lymph node stroma) (**e**) of the aortic necrotic core area in the NC, *Ldlr*^−/−^, flox and CKO groups (*n* = 6 per group). *P < 0.05. **f**,**g**, Flow cytometry analysis of immune cells isolated from atherosclerotic plaques of the flox and CKO groups (*n* = 6 per group), showing the CTL ratio (**f**) and the ratio of activated CTLs (defined as PD1^+^CD107a^+^) (**g**). *P < 0.05.

To delineate the role of Kdm6b in macrophage-induced atherosclerosis, we generated macrophage-specific *Kdm6b*-knockout mice on an LDL receptor-deficient (*Ldlr*^−/−^) background and used *Csf1r*-cre to drive recombination in macrophages in *Kdm6b*-floxed mice. Heart function and major biochemical indicators, with the exception of triglycerides, were comparable among the four groups (Supplementary Fig. 7a,b). En face aorta staining and histological evaluation with hematoxylin and eosin revealed a reduction in the atherosclerotic lesion area in the CKO group compared with both the *Ldlr*^−/−^ group and the flox control group (Fig. 7a,b). Furthermore, we observed a decrease in the accumulation of collagen fibers (Fig. 7c,d) and in macrophage infiltration (Fig. 7e) in the CKO group. To characterize the phenotype of CTLs within atherosclerotic plaques further, we isolated the plaques and conducted cytometric analysis. As depicted in Fig. 7f,g, both the total ratio and the activation status of CTLs decreased after the macrophage-specific knockout of *Kdm6b*. Interestingly, systemic use of m^6^A modification inhibitors also delayed plaque formation, collagen accumulation and macrophage and CTL infiltration (Supplementary Fig. 8a–h). These findings suggest that KDM6B in macrophages contributes to the progression of atherosclerosis partly by promoting the activation of CTLs and IFN-γ-dependent macrophage activation.

## Discussion

m^6^A is recognized as the most prevalent posttranscriptional modification of mRNAs and is known to regulate various aspects of macrophage function, including development, activation, inflammation^12,13^, polarization^29^, aging^30^, pyroptosis, lipid uptake^31^ and cholesterol efflux^32^. Atherosclerosis is a chronic inflammatory disease driven by lipid accumulation and immune dysregulation. METTL3, a m^6^A writer, is upregulated in M1 macrophages within atherosclerotic plaques. It stabilizes hepatoma-derived growth factor mRNA via m^6^A modification, reprogramming energy metabolism to increase glycolysis and suppress mitochondrial respiration, which promotes plaque progression^29^. However, the increase in Mettl3-mediated AKT1S1 reduces foam cell formation by activating macrophage autophagy^33^. These studies revealed the bidirectional regulatory role of METTL3 following atherosclerosis progression. Moreover, knockout of Mettl14, another m^6^A writer, significantly reduces the inflammatory response of macrophages by decreasing *Myd88* mRNA stability and the development of atherosclerotic plaques^34^. Genome-wide analyses revealed m^6^A peaks enriched in coding sequences (CDSs) and 3′ untranslated regions (UTRs) of macrophage polarization-related genes (for example, STAT6 and PI3K/AKT pathway components), with consensus DRACH motifs^35^. The above studies indicate that m^6^A modifications in macrophages are strongly associated with atherosclerosis. Despite these insights, systematic studies tracking the dynamic changes in m^6^A modifications throughout macrophage differentiation and activation are lacking. To fill this gap, we conducted meRIP-seq on monocyte-derived and IFN-γ-stimulated macrophages in this study. Integrated analysis with RNA-seq revealed that, during macrophage differentiation, pathways involved in inflammation, innate immunity, small molecule metabolism and protein processing are extensively regulated by m^6^A modification. After differentiation, an increase in the number of mRNAs containing m^6^A modifications highlights its pivotal role. Furthermore, a cluster of genes, including myeloid cell markers and immunological factors, continued to be modulated by m^6^A. However, during macrophage activation, cytokine- and inflammation-related genes are positively regulated by m^6^A. These findings highlight the precise and stage-specific regulation of m^6^A modifications on mRNAs during macrophage differentiation and activation. The molecular machinery governing this regulation remains elusive and merits further investigation.

The JAK–STAT pathway is a critical signaling cascade that orchestrates macrophage polarization and inflammatory responses. Upon the binding of cytokines (for example, IFN-γ and IL-6) to their receptors, JAK kinases (JAK1, JAK2 and TYK2) are activated and phosphorylate STAT1. Phosphorylated STAT1 (p-STAT1) dimerizes, translocates to the nucleus and drives the transcription of proinflammatory genes, including IRF1, STAT1 itself, CXCL9/10 and NOS2 (inducible nitric oxide synthase, iNOS), thereby promoting M1-like macrophage polarization^36^. Persistent STAT1 activation in M1 macrophages drives plaque instability in atherosclerosis^37^. Meanwhile, activation of cytokine signaling (for example, via the IL-6–gp130 complex, CSF2 (GM-CSF) or oncostatin M receptor) had been reported to phosphorylate STAT3, driving macrophages toward an M2-polarized phenotype in multiple cancers^38–40^. Conversely, application of a STAT3 inhibitor has been shown to alleviate atherosclerosis by suppressing endothelial dysfunction, macrophage differentiation and CD4^+^ T effector activation^41^. In this study, our group demonstrated that the JAK–STAT1 pathway is regulated by the histone demethylase KDM6B and connects this epigenetic enzyme with JAK–STAT1 signaling in atherosclerotic macrophages. Moreover, the unspecific role of KDM6B during JAK–STAT1 activation was interpreted, which contributes to the understanding of the nonhistone demethylation activities of KDM6B. However, the overall process by which KDM6B demethylates JAK1 needs to be further explored.

Furthermore, during atherosclerosis progression, environmental factors such as oxidized lipids, inflammatory cytokines and cholesterol crystals also induce epigenetic heterogeneity in macrophages^42^. The histone demethylase KDM6B has been implicated in NF-κB-mediated inflammatory activation of macrophages in abdominal aortic aneurysms^7^. Interestingly, myeloid cell-specific deficiency of Kdm6b has been associated with the promotion of advanced atherosclerotic plaques^43^, a finding that contrasts with our results. In our opinion, three factors may contribute to these discrepancies: (1) Kdm6b deletion changes monocyte-derived macrophage differentiation and subsequent myeloid cell subset infiltration; (2) the interaction protein spectrum of Kdm6b in monocytes and macrophages differs, resulting in differential interaction targets during IFN-γ stimulation; and (3) cell type-specific Kdm6b deletion changes the interaction relationship between myeloid cells and other immune cells or stromal cells. More experiments should be carried out on myeloid cell subset-specific Kdm6b deletion mice to elucidate the reasons. In addition, KDM6B mRNA regulation by m^6^A modification was observed during macrophage activation but not during differentiation. However, KDM6B is crucial for the differentiation of monocytes into macrophages, which suggests that myeloid cell-specific KDM6B deficiency might impair this process.

Our research presents a comprehensive mapping of the m^6^A modification landscape throughout macrophage differentiation and activation for the first time. We revealed that m^6^A modification plays a pivotal role in the upregulation of KDM6B in response to IFN-γ stimulation, which is essential for the phosphorylation of STAT1.

## Supplementary information

Supplementary Information

## Data Availability

Raw RNA-seq and RIP-seq data were deposited in NCBI under the accession numbers GSE271963, GSE274228 and GSE274511. All original data used for this study are available from the corresponding author upon reasonable request.
