# Supplementary material for: METTL3/RBM15 augments the stability of Kdm6b mRNA and promotes STAT1-mediated macrophage activation and atherosclerosis
Source: Exp Mol Med. 2025 Dec 22;57(12):2916–29. doi: 10.1038/s12276-025-01594-y (PMC12800227; doi:10.1038/s12276-025-01594-y)
Supplement: Supplementary file 1 — Supplementary Information [file 12276_2025_1594_MOESM1_ESM.pdf]

Supplementary Information

METTL3/RBM15 augments the stability of Kdm6b mRNA and promotes STAT1-mediated macrophage activation and atherosclerosis

Ning Huangfu[1,2,3*], Fang Li[4,5*], Chenqiu Wang[4,5*], Shouyi Jin[4,5], Xiaoya Zheng[1], Yingsong Wang[1], Tianxiang Fang[1], Jiaxi Shen[1,2,3], Yanan Yu[6], Liguo Jian[4,5#], Datun Qi[6#], Wenting Zhao[7#], and Dongdong Jian[6#].

[1]Department of Cardiology, The First Affiliated Hospital of Ningbo University, Ningbo, 315010, China.

[2]Department of Cardiology, Key Laboratory of Precision Medicine for Atherosclerotic Diseases of Zhejiang Province, Ningbo, 315010, China.

[3]Clinical Medicine Research Centre for Cardiovascular Disease of Ningbo, Ningbo, 315010, China.

[4]Department of Cardiology, The Second Affiliated Hospital of Zhengzhou University, Zhengzhou, Henan, 450014, China.

[5]Tianjian Laboratory of Advanced Biomedical Sciences, Institute of Advanced Biomedical Sciences, Zhengzhou University, Zhengzhou, Henan, 450001, China.

[6]Zhengzhou Key Laboratory of Cardiovascular Aging, Henan Province Key Laboratory for Prevention and Treatment of Coronary Heart Disease, National Health Commission Key Laboratory of Cardiovascular Regenerative Medicine, Central China Fuwai Hospital of Zhengzhou University, Fuwai Central China Cardiovascular Hospital & Central China Branch of National Center for Cardiovascular Diseases, Zhengzhou, Henan, 451450, China.

[7]The First Affiliated Hospital, Zhejiang University School of Medicine. Hangzhou, 310003, China.

[*]These authors contribute equally.

Corresponding authors: jdd1102@zzu.edu.cn (D.J.); 1320002@zju.edu.cn (W.Z.); qimedicine@zzu.edu.cn (D.Q.) and sahjlg@zzu.edu.cn (L.J.).

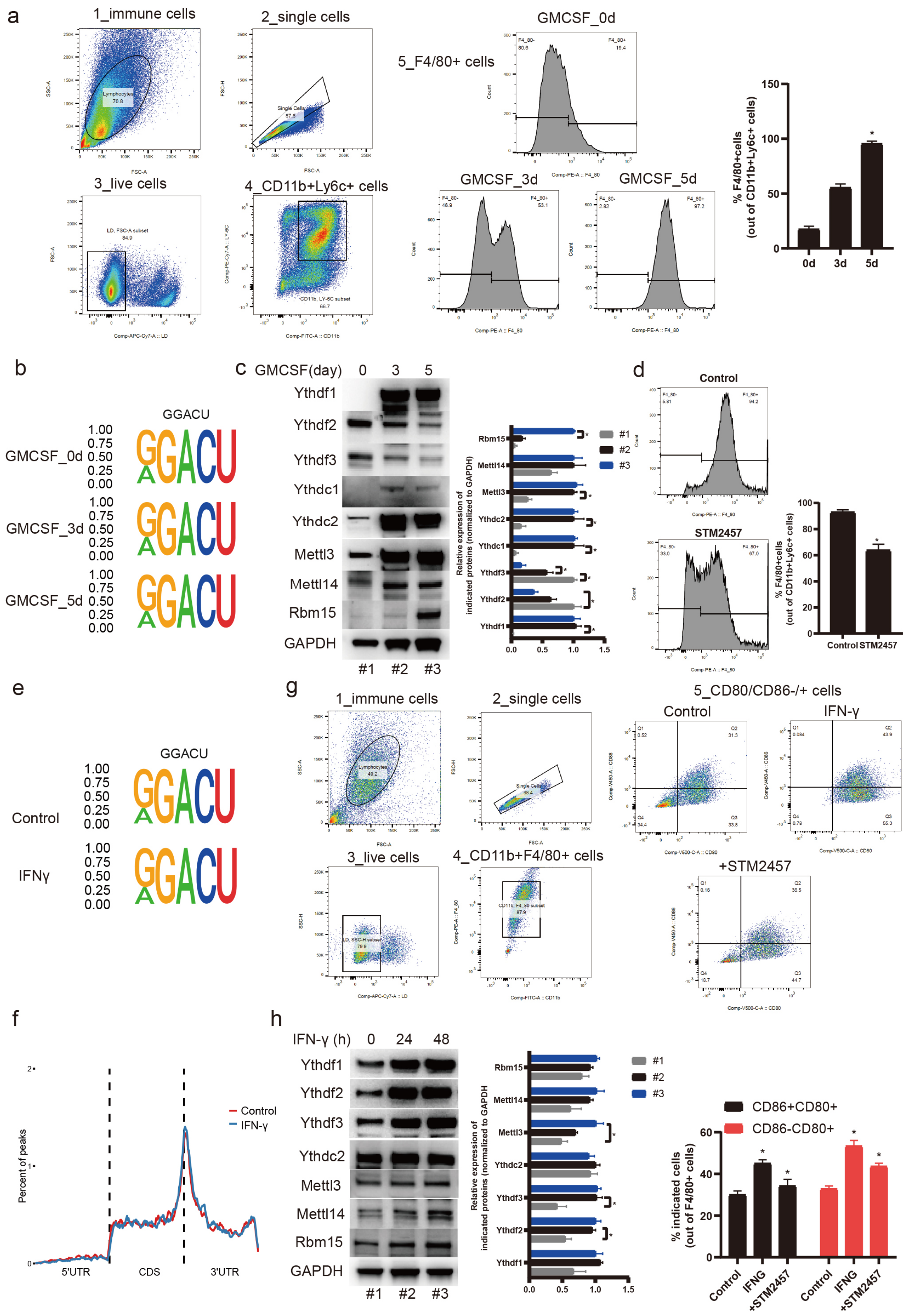

Supplementary Fig. 1. Flow cytometry gating strategy and m6A modification is necessary for macrophage differentiation and activation.

a: Monocytes were isolated and stimulated with GM-CSF to induce their differentiation into macrophages. The F4/80+ cells among the CD11b+Ly6c+ cells were recognized as macrophages.

b: Motif analysis of m^6A-RIP-seq data from GM-CSF-treated monocytes.

c: Monocytes were stimulated with GM-CSF at the indicated time points. m^{6}A modification-related factors were detected using western blotting.

d: Monocytes were treated with GM-CSF or GM-CSF and STM2457, and the F4/80+ cell ratio was detected using flow cytometry.

e: Motif analysis of m6A-RIP-seq data from IFNγ-treated macrophages.

f: Distribution of m6A peaks in gene body regions, including the 5’UTR, CDS and 3’UTR.

g: Macrophages were stimulated with IFN-γ, and the CD80+CD86+ cell ratio was detected and recognized as activated macrophages.

h: Macrophages were treated with IFN-γ at the indicated time points. m^{6}A modification-related factors were detected using western blotting.

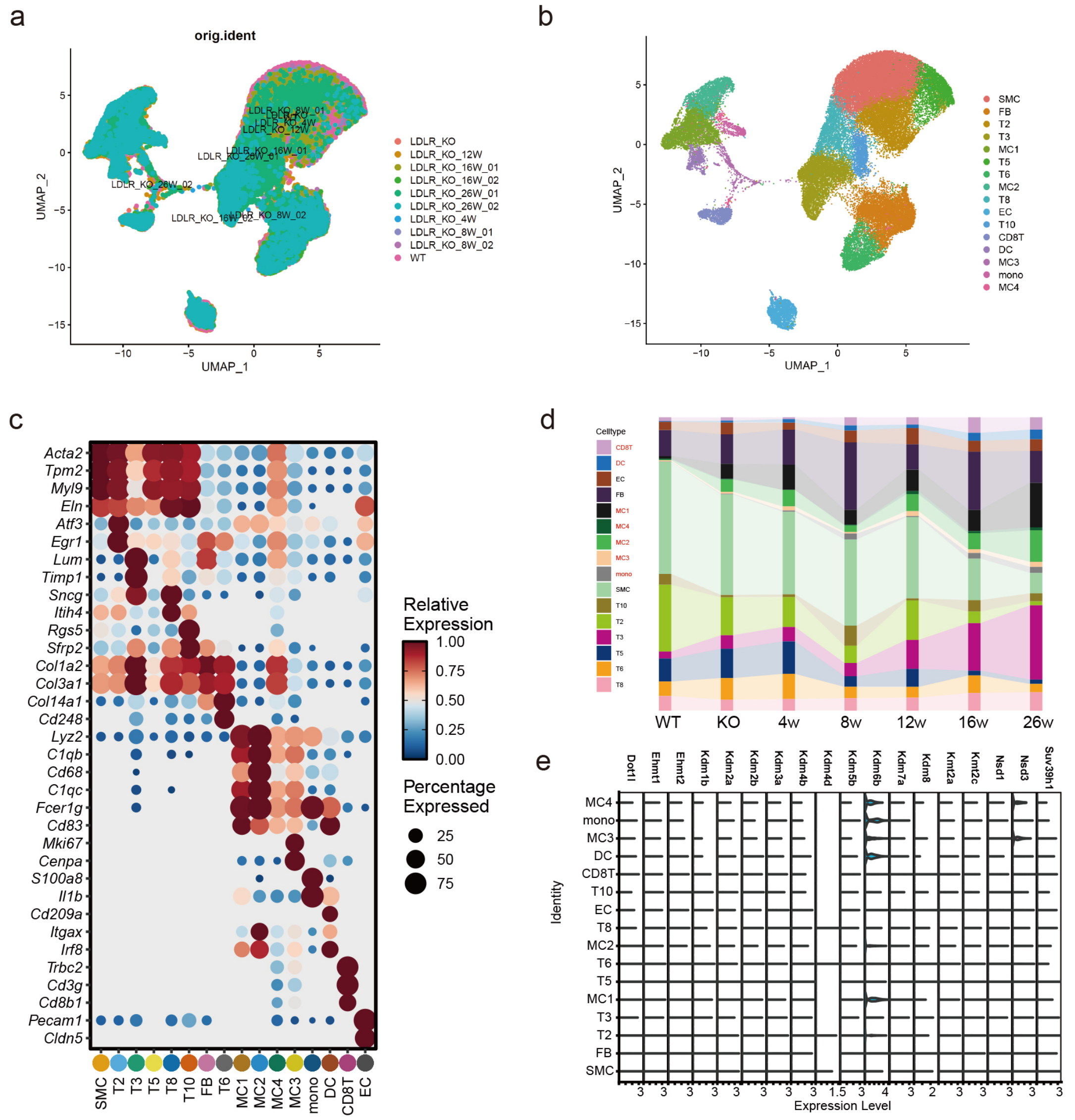

Supplementary Fig. 2. Kdm6b is specifically expressed in myeloid cell clusters.

a-b: UMAP plot showing the sample (a) or cluster (b) distributions of mouse atherosclerotic plaque via scRNA-seq.

c: Heatmap showing the expression of marker genes of cell clusters from scRNA-seq.

d: Sankey diagram showing the ratio of each cluster across seven groups.

e: Violin plot showing the expression of the indicated epigenetic enzyme mRNAs across different cell subsets isolated from atherosclerotic plaques in mice.

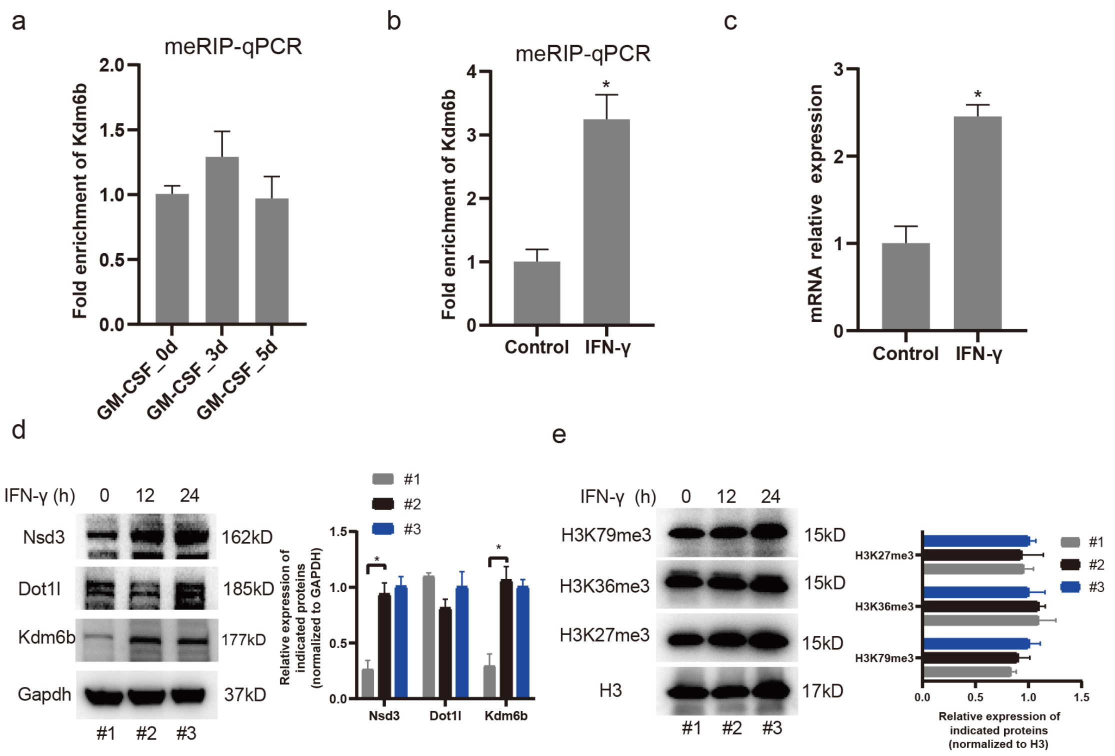

Supplementary Fig. 3. IFN-γ induces Kdm6b expression.

a-b: Monocytes were stimulated with GM-CSF (a) or IFN-γ (b) to induce macrophage differentiation or activation. MeRIP-qPCR was subsequently performed. KDM6B mRNA levels were quantified by real-time qPCR. $^{*}P < 0.05$.

c: Macrophages were treated with IFN-γ for 12 h. The mRNA levels of KDM6B were measured by quantitative qPCR. $^{*}P < 0.05$.

d-e: Monocytes were stimulated with IFN-γ for the indicated durations. Western blot detection of NSD3, DOT1L, KDM6B, H3K79me3, H3K36me3, and H3K27me3 in macrophages. GAPDH and H3 served as internal controls for cytoplasmic and nuclear proteins, respectively.

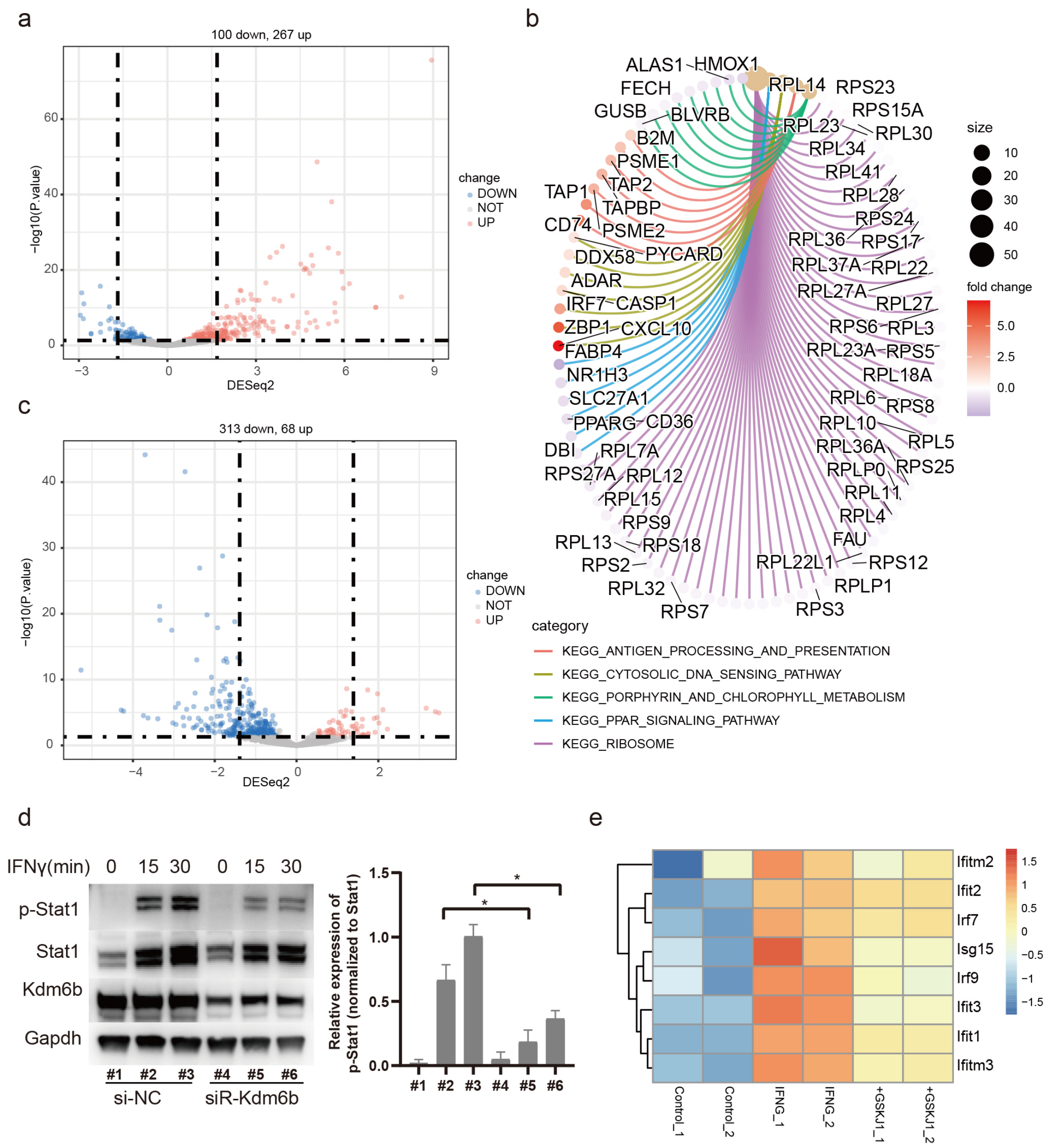

Supplementary Fig. 4. GSKJ1 suppresses IFN-III signaling.

a: Volcano plot showing the differential gene expression between the control group and the IFNγ group.

b: Cnet plot showing enriched KEGG pathways identified from differentially expressed genes between the control macrophages and the IFNγ-treated macrophages.

c: Volcano plot showing the differential gene expression between the IFNγ group and the IFNγ+GSKJ1 groups.

d: Macrophages were pretransfected with siR-Kdm6b for 24 h and stimulated with IFN-γ at the indicated times. Western blot analysis of Stat1, phosphorylated Stat1 (p-Stat1), and Kdm6b was performed, with Gapdh used as an endogenous control.

e: Heatmap showing differentially expressed IFN-γ-targeted genes among the control, IFNγ and GSKJ1+ IFNγ groups.

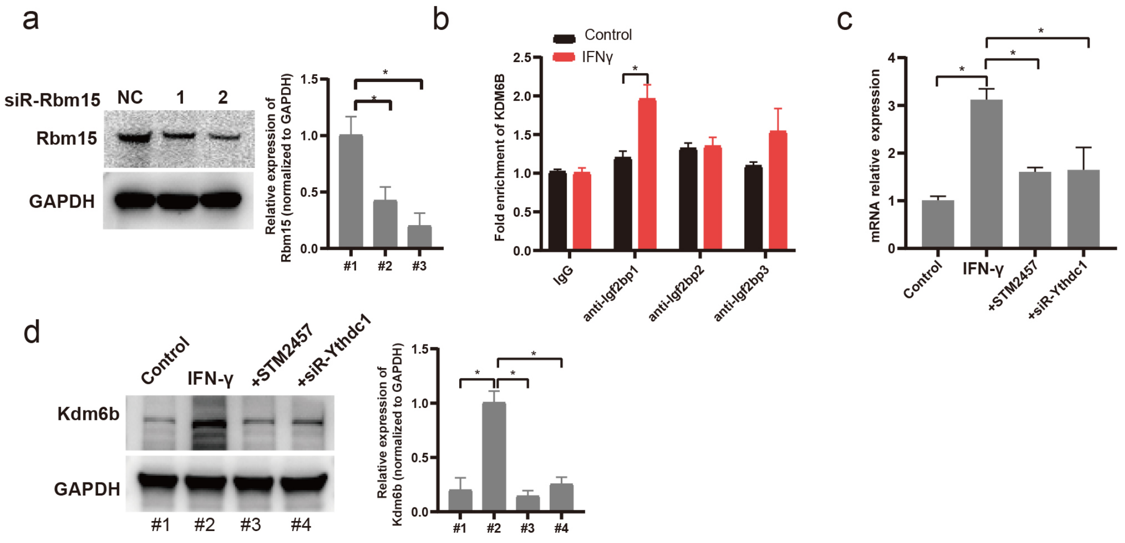

Supplementary Fig. 5. m6A modification factors control Kdm6b expression.

a: Macrophages were transfected with siRNAs against Rbm15 for 48h. Rbm15 protein expression was detected using western blotting.

b: Macrophages were stimulated with IFN-γ for 12 hours. A RIP assay was performed to assess the interaction between Kdm6b mRNA and the indicated antibodies in macrophages stimulated with IFNγ. $^{*}P < 0.05$.

c: Macrophages were stimulated with IFN-γ for 12 hours. qPCR was performed to assess the expression of Kdm6b upon STM2457 treatment or Ythdc1 knockdown. $^{*}P < 0.05$.

d: Macrophages pretreated with STM2457 or transfected with siR-Ythdc1 were stimulated with IFN-γ for 24 hours. Kdm6b protein expression was detected using western blotting.

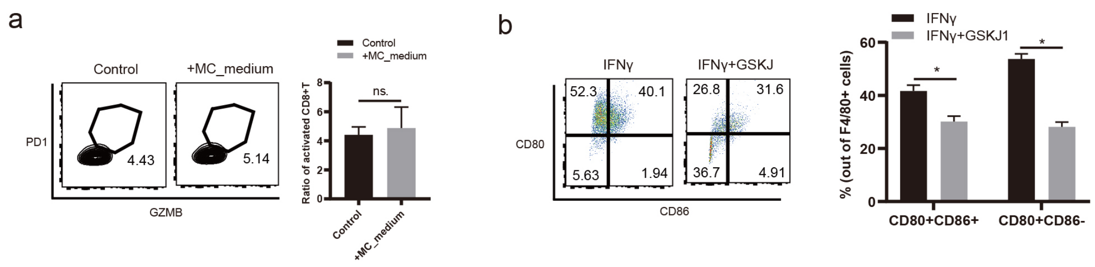

Supplementary Fig. 6. GSKJ1 inhibits macrophage activation.

a: CTLs were cocultured with medium from macrophages for 24 hours. Flow cytometry detection of the activated CTL ratio.

b: Macrophages were pretreated with GSKJ1 for 30 min and stimulated with IFN-γ for 24 h. The CD80+CD86+ cell ratio among F4/80 cells was recognized as the number of activated macrophages, as detected by flow cytometry. $^{*}P < 0.05$.

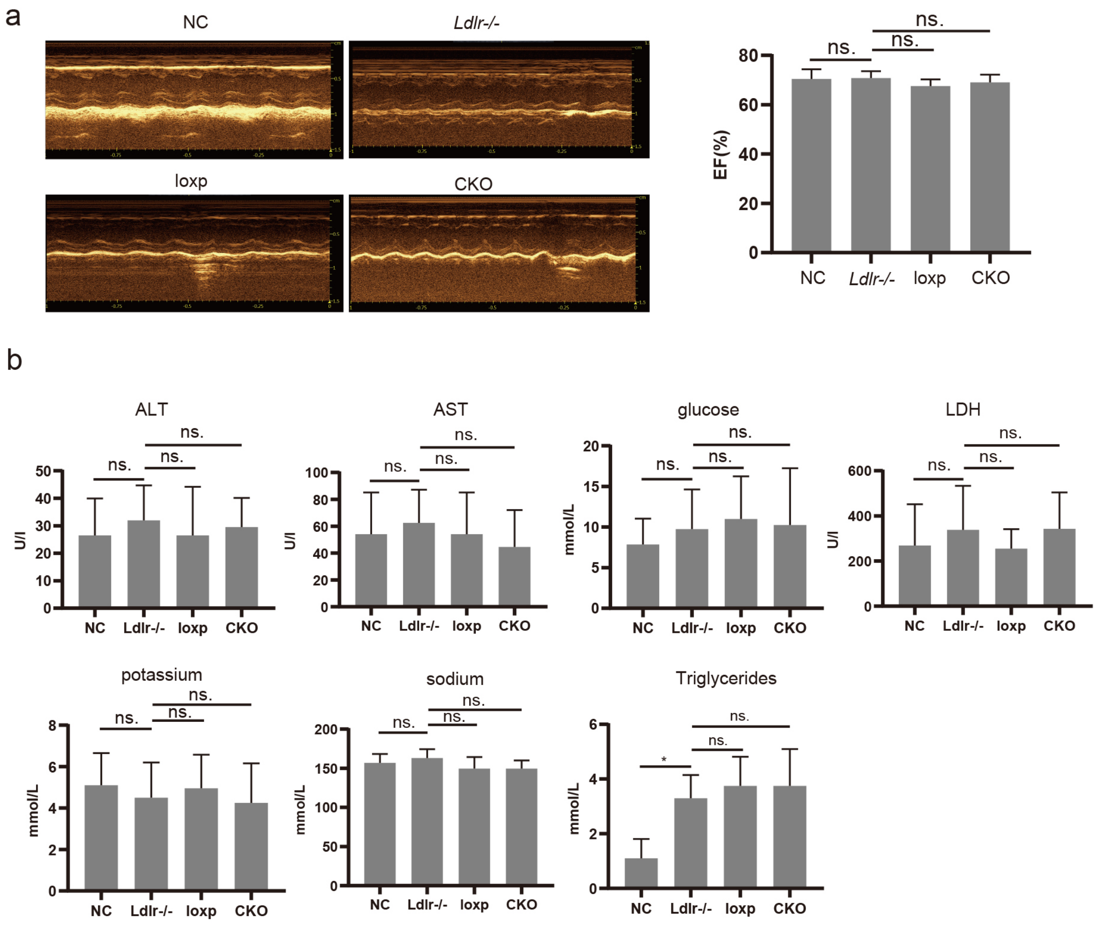

Supplementary Fig. 7. Triglycerides are induced in *Ldlr-/-* mice compared to wild type mice.

a: Echocardiograms evaluation of cardiac function was carried out in parasternal long-axis windows as shown in the representative 2D example (left). Right panel shows the quantified results of ejection fraction (EF) values of mice at 8 weeks of age in the indicated groups. n=6 for each group. ns, no significance.

b: Detection of serum biochemical indicators (ALT, AST, glucose, LDH, potassium, and sodium) in mice under baseline conditions and triglyceride levels in mice at 8 weeks of age. ALT, Alanine Aminotransferase; AST, Aspartate Aminotransferase; LDH, Lactate Dehydrogenase. ns, no significance. n=6 for each group. ns, no significance. $^{*}P < 0.05$.

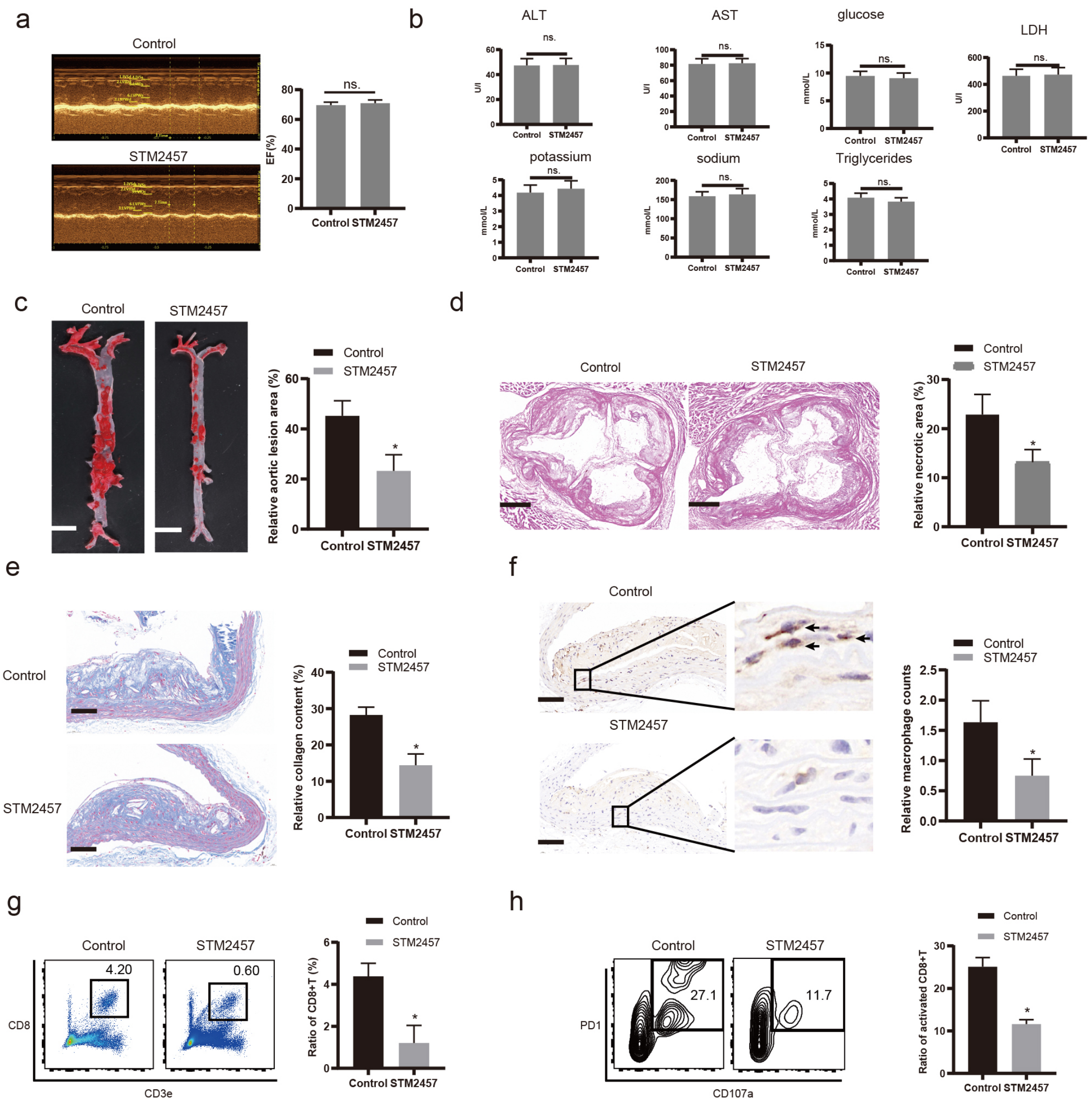

Supplementary Fig. 8. Inhibition of m6A modification delays atherosclerosis development.

Ldlr-/- mice were fed a HFD (Control group) or a HFD supplemented with STM2457 (STM2457 group) for 16 weeks.

a: Echocardiograms evaluation of cardiac function was carried out in parasternal long-axis windows as shown in the representative 2D example (left). Right panel shows the quantified results of ejection fraction (EF) values in the indicated groups. n=6 for each group. ns, no significance.

b: Mice biochemical indicators in serum were analyzed using ADVIA 2120i system.n=6 for each group. ns, no significance.

c: Representative images and quantification of Oil Red O staining of aorta en face lesions isolated from the control and STM2457 groups. n = 6 per group. $^{*}P< 0.05$.

d: H&E staining images and quantification of the necrotic core area in aortic sections from the control and STM2457 groups. n = 6 per group. $^{*}P < 0.05$.

e-f: Histological analysis with Masson's trichrome staining (e) and MOMA2 (macrophage/monocyte monoclonal antibody) staining (antigen: mouse lymph node stroma) (f) of the aortic necrotic core areas of the control and STM2457 groups. n = 6 per group. $^{*}P < 0.05$.

g-h: Flow cytometry analysis of immune cells isolated from atherosclerotic plaques of the control and STM2457 groups (n = 6 per group). Panel g shows the CTL ratio, while panel h displays the ratio of

activated CTLs (defined as PD1+CD107a+). $^{*}P < 0.05$.
